# Experimental models to study oxidative stress in glaucoma: integrating *in vitro*, *in vivo*, and clinical studies

**DOI:** 10.3389/fphar.2026.1893993

**Published:** 2026-08-18

**Authors:** Laxmi Moksha, Yan Zhu, Rebecca Salowe, Vrathasha Vrathasha, Rohini M. Nair, Jie He, Mina Halimitabrizi, Gabriella Rice, Peter M. J. Quinn, Yuyan Cheng, Ahmara G. Ross, Bruce Ksander, Joan M. O’Brien

**Affiliations:** 1 Center for Genetics of Complex Disease, Department of Ophthalmology, Scheie Eye Institute, University of Pennsylvania, Philadelphia, PA, United States; 2 F.M. Kirby Center for Molecular Ophthalmology, University of Pennsylvania, Philadelphia, PA, United States; 3 Department of Neurology, University of Pennsylvania, Philadelphia, PA, United States; 4 Schepens Eye Research Institute of Massachusetts Eye and Ear, Boston, MA, United States; 5 Department of Ophthalmology, Harvard Medical School, Boston, MA, United States

**Keywords:** glaucoma, neurodegeneration, oxidative stress, reactive oxygen species, retinal ganglion cells

## Abstract

Glaucoma is a complex optic neuropathy and the leading cause of permanent blindness worldwide. Elevated intraocular pressure (IOP) is the major modifiable risk factor for this disease. Growing evidence suggests that oxidative stress and reactive oxygen species (ROS) are key mediators of retinal ganglion cell (RGC) death and optic nerve (ON) damage. In this review, we summarize experimental models that are routinely used to study oxidative stress in glaucoma and discuss the translational value of each model system for biomarker discovery and treatment development. This narrative review focuses on studies that emphasize experimental and clinical models relevant to oxidative stress in glaucoma. Primary experimental and clinical studies that directly investigated pathways of oxidative stress in glaucoma models were included for contextual synthesis. *In vitro* systems such as TM cells, RGCs, and induced pluripotent stem cell (iPSC)-derived models enable controlled conditions to study cellular processes of ROS-induced stress and to screen potential therapeutic agents. Three-dimensional culture systems provide even more physiological insights by replicating retinal development, organization, and function. *In vivo* models include microbead-induced ocular hypertension (OHT), episcleral vein cauterization (EVC), and genetic models, allowing for the study of oxidative stress in the context of raised IOP, neuroinflammation, and vascular dysregulation. Findings from human donor tissues and clinical biospecimens provide translational support for experimental models by showing oxidative damage in glaucomatous eyes. Across model systems, oxidative stress is associated with glaucoma-related cellular and molecular changes, including mitochondrial dysfunction, neuroinflammation, synaptic instability, and altered neurotrophic signaling. This review provides a structured, model-based overview of how oxidative stress can be investigated across *in vitro*, *in vivo*, and clinical systems for glaucoma research. Although it is not intended to be fully comprehensive, it highlights key experimental platforms that can guide model selection for studying ROS-mediated mechanisms and developing neuroprotective strategies.

## Graphical Abstract

**Figure:**
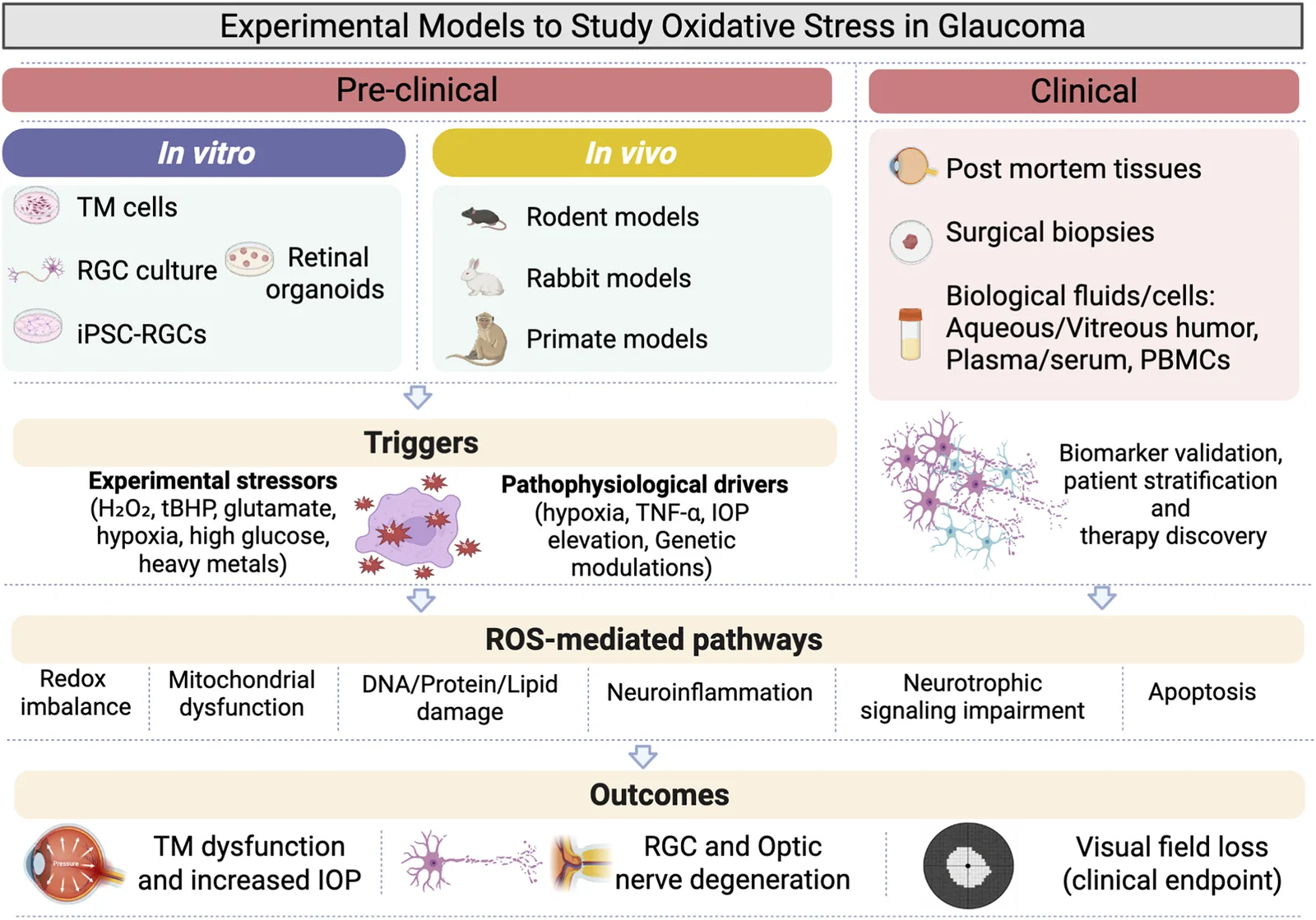

## 1 Introduction

Glaucoma is a heterogeneous group of ocular disorders, but all subtypes are characterized by progressive optic nerve (ON) degeneration marked by retinal ganglion cell (RGC) loss, retinal nerve fiber layer (RNFL) thinning, and optic disc cupping (Weinreb et al., 2014; Schuster et al., 2020; Stein et al., 2021). The disease is the leading cause of irreversible blindness globally and typically begins with peripheral vision loss, making it difficult to diagnose in early stages (Stein et al., 2021). By 2040, glaucoma will affect 111.8 million people worldwide (Tham et al., 2014). Up to 50% of glaucoma patients may present without elevated intraocular pressure (IOP), a pattern often referred to as normal-tension glaucoma (NTG). This suggests that vascular, metabolic, mitochondrial, immune, and neurodegenerative mechanisms may also contribute to disease pathophysiology (Anderson, 2011; Weinreb et al., 2014).

According to many studies, the development of glaucoma can be traced back to events associated with microcirculation, mitochondria, immunity, excitotoxicity, oxidative stress, and systemic effects (Berdahl, Allingham, and Johnson, 2008; Ju et al., 2023). Several glaucoma-associated genes and risk loci affect either trabecular meshwork (TM) function or RGC vulnerability. Myocilin (*MYOC)* is mainly linked to TM dysfunction and impaired aqueous humor (AH) outflow*,* whereas *OPTN, TBK1*, *CDKN2B-AS1*, *SIX6, TXNRD2,* and *ABCA1* are more closely associated with RGC vulnerability, mitochondrial stress, autophagy, senescence, and oxidative injury pathways (Wiggs et al., 2012; Bailey et al., 2016; Wiggs and Pasquale, 2017; Gharahkhani et al., 2021). *MYOC, OPTN, TBK1,* and *CDKN2B-AS1* are glaucoma risk loci with tissue-specific functions. *MYOC* mutations cause TM malfunction and reduced aqueous outflow, whereas *OPTN* and *TBK1* cause RGC degeneration via mitochondrial failure and autophagy (Joe et al., 2003; Tucker et al., 2014; Fingert et al., 2016; Kasetti et al., 2016; Shim et al., 2016; Zhang et al., 2021).

Among the various contributing mechanisms, oxidative stress (Tezel, 2006) is increasingly recognized as a key mechanism contributing to RGC dysfunction and death in glaucoma. In the anterior segment, ROS can affect TM cell survival, extracellular matrix (ECM) remodeling and AH outflow resistance, thereby contributing to IOP elevation (Xu et al., 2025; Saccà and Izzotti, 2008; Wang et al.*,* 2019). Concurrently, in the posterior segment, excessive accumulation of ROS in ocular tissues disrupts mitochondrial homeostasis and induces oxidative damage to DNA, proteins, and lipids, thereby promoting RGC loss and ON degeneration (Bugara et al., 2024). ROS-mediated injury has also been linked to ferroptosis, impaired axonal transport, and activation of pro-inflammatory signaling pathways (Nickells et al., 2012; Weinreb et al., 2014; Hurley et al., 2022; Zhou and Liu, 2026). Because glaucomatous damage spans early outflow-pathway dysfunction and chronic RGC and ON degeneration, different experimental models are required to address distinct disease stages and mechanisms.

Although ROS-mediated injury is increasingly recognized as an important contributor to glaucoma, no single experimental model fully reproduces the temporal, cellular, and molecular complexity of the human disease (Johnson and Tomarev, 2010; Bouhenni et al., 2012; Harada et al., 2020; Fan Gaskin et al., 2021). Oxidative stress is involved in the entire cascade of the disease, and experimental models are needed to uncover pathways and to understand how they influence the evolution of glaucoma. Here, we review ROS-driven glaucoma pathophysiology using a model-based framework that includes *in vitro* systems, *in vivo* models, and human clinical samples. We underline that different experimental models are appropriate for different oxidative stress triggers and phases of glaucomatous damage and that the selection of the system should be driven by mechanistic issues. This review describes experimental oxidative stress pathways, but it is not exhaustive. Its primary purpose is to aid selection of models based on the research topic, biological endpoint, disease stage, and translational goals.

Accumulating evidence suggests that early glaucomatous injury affects neuronal function before overt cell loss occurs. Synaptic dysfunction is a common early feature of this process, and RGCs may show dendritic retraction, reduced expression of synaptic proteins such as postsynaptic density protein 95 (PSD-95), and disrupted interneuron-RGC connectivity before measurable soma loss or axonal degeneration occurs (Williams et al., 2013; Bugara et al., 2024). Oxidative stress may contribute to this synaptic instability by impairing mitochondrial function and promoting inflammatory responses that compromise synaptic integrity (Ju et al., 2023).

Neurotrophic pathways are affected early in glaucoma. Glaucomatous stress increases RGC sensitivity via impairment of the neurotrophin system, particularly the BDNF/TrkB axis (Kimura et al., 2016). In animal models of ocular hypertension (OHT), axonal transport failure has been associated with RGC trophic deprivation due to altered retinal expression and defective retrograde transport of neurotrophins and receptors (Rudzinski, Wong, and Uri Saragovi, 2004; Gupta et al., 2014; Hurley et al., 2022).

**FIGURE 1:**
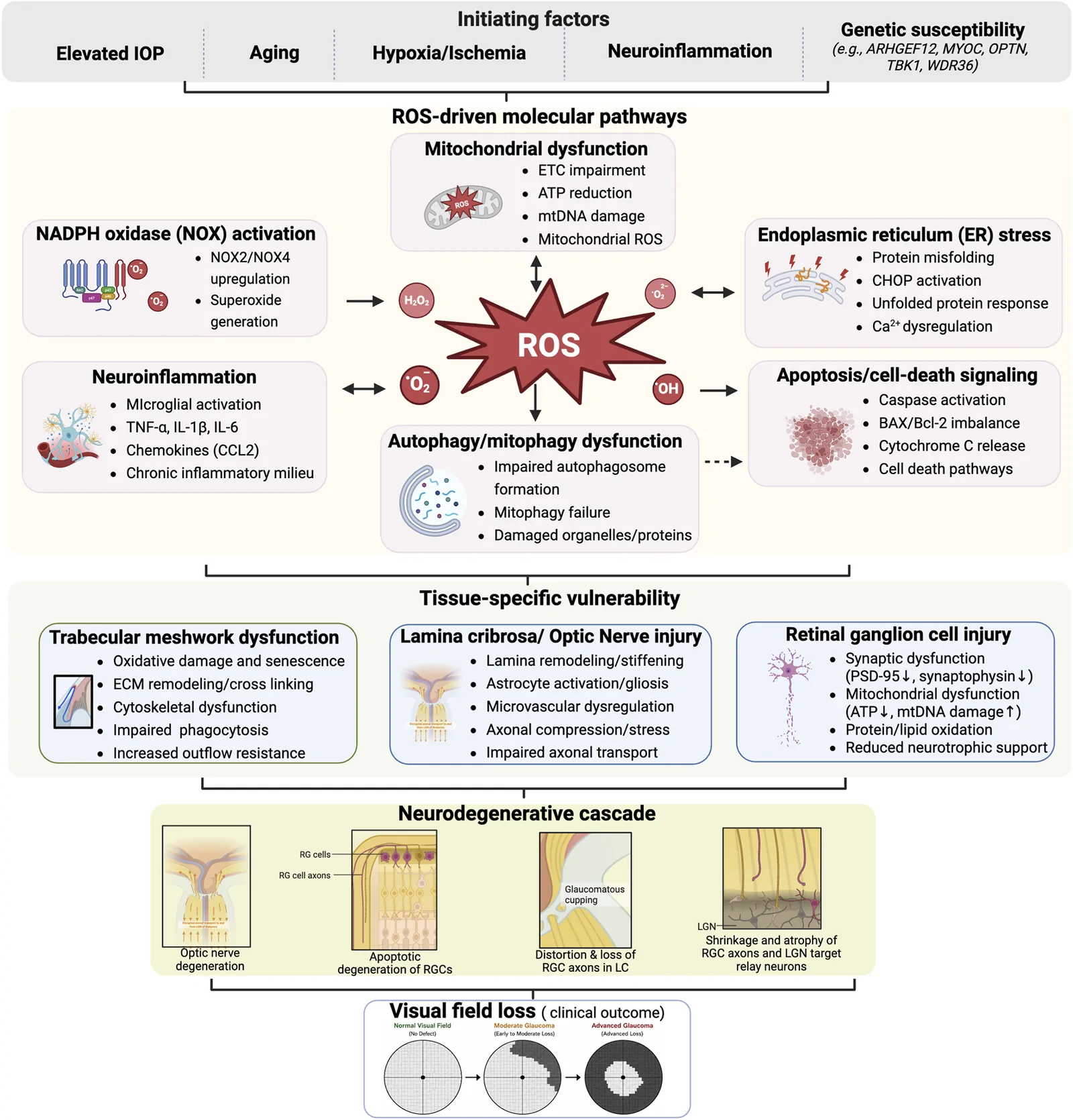
Mechanistic overview of oxidative stress-driven glaucomatous neurodegeneration. This schematic illustrates how elevated intraocular pressure, aging, hypoxia/ischemia, neuroinflammatory triggers, and genetic susceptibility can contribute to oxidative stress-related pathways in glaucoma. These pathways include NADPH oxidase activation, mitochondrial dysfunction, endoplasmic reticulum stress, neuroinflammation, impaired autophagy/mitophagy, and apoptosis/cell-death signaling. Tissue-specific vulnerability in the trabecular meshwork, lamina cribrosa/optic nerve head, and retinal ganglion cells can impair aqueous humor outflow, disrupt axonal transport, reduce neurotrophic support, promote retinal ganglion cell injury, and contribute to optic nerve degeneration and progressive visual field loss. Abbreviations: ROS, reactive oxygen species; IOP, intraocular pressure; NOX, NADPH oxidase; RGC, retinal ganglion cell; TM, trabecular meshwork; ER, endoplasmic reticulum; ETC, electron transport chain; ECM, extracellular matrix; mtDNA, mitochondrial DNA. Created with BioRender.

Figure 1 provides a schematic overview of how major glaucoma-related stressors, including elevated IOP, aging, hypoxia/ischemia, neuroinflammatory triggers, and genetic susceptibility, converge on oxidative stress-related pathways. These pathways include NOX activation, mitochondrial dysfunction, endoplasmic reticulum (ER) stress, neuroinflammation, impaired autophagy/mitophagy, and apoptosis or other cell-death signaling. Together, these processes affect the TM, lamina cribrosa/ON head, and RGCs, contributing to impaired AH outflow, axonal transport disruption, reduced neurotrophic support, RGC injury, ON degeneration, and progressive visual field loss. The inclusion of neurotrophic failure and synaptic dysfunction is consistent with evidence implicating disrupted BDNF/TrkB signaling in glaucomatous RGC vulnerability and supports neuroprotective strategies beyond IOP lowering alone (Kimura et al., 2016; Mallone et al., 2020). Together, these processes contribute to tissue-specific consequences in the TM, ON head, lamina cribrosa, and RGCs, leading to ON degeneration, impaired axonal transport, neurotrophic failure, RGC dysfunction, and progressive visual field loss. This narrative review emphasizes representative oxidative stress-related pathways in experimental systems and is not intended to be exhaustive. Its main objective is to guide model selection according to the research question, biological endpoint, disease stage, and translational purpose.

## 2 *In vitro* models

Mechanistic investigations of oxidative signaling pathways and high-throughput drug screening are frequently performed using in vitro systems because oxidative stress levels can be precisely controlled.

### 2.1 Models of oxidative stress induction

Several methods are used to induce oxidative stress in experimental glaucoma models. Methods that are commonly used include hypoxia/reoxygenation, glutathione depletion, and chemicals that generate ROS such as hydrogen peroxide (H_2_O_2_) and tert-butyl hydroperoxide (tBHP) (Saccà and Izzotti, 2014; Vernazza et al., 2019; Ransy et al., 2020; Abd Alhadi et al., 2025). Cueva Vargas et al. (2015) found that tumor necrosis factor alpha (TNF-α) activates redox-sensitive inflammatory pathways in glaucoma, which in turn produce oxidative stress. This finding emphasizes the link between inflammation and ROS. Because they are easy to use, reproducible, and fast at causing intracellular oxidative stress, chemical ROS inducers such as H_2_O_2_ and tBHP are widely used in experimental studies. Each of these approaches provides insights into mechanisms underlying TM and RGC dysfunction. The benefits and drawbacks of these techniques are compared in Table 1. These few induction techniques will be followed by other models of oxidative stress. With the help of the oxidative stress inducer, models can mimic inflammatory oxidative signaling, mitochondrial dysfunction, or acute or chronic ROS exposure. In general, oxidative stress induction methods *in vitro* provide regulated and reproducible systems to unravel ROS-mediated processes, although their translational value is dependent on how well they mimic physiological stress conditions.

**TABLE 1:** Experimental approaches used to induce oxidative stress in glaucoma models.

Oxidative stress inducer	Mechanistic basis	Major pathways involved	Common application	Key advantages	Key limitations	Representative references
H_2_O_2_	Directly increases intracellular reactive oxygen species, causing oxidative damage to lipids, proteins, DNA, and mitochondria	NF-κB signaling; MAPK/ERK activation; mitochondrial apoptotic pathways	Mainly *in vitro* models, including trabecular meshwork (TM) cells and retinal ganglion cell (RGC) cultures	Simple, reproducible, cost-effective, and dose-dependent	Represents an acute oxidative insult and may not fully reproduce chronic oxidative stress in glaucoma	Hong et al. (2021), Verma et al. (2024)
tBHP	Promotes lipid peroxidation and mitochondrial reactive oxygen species generation, leading to membrane damage and ferroptosis-like cell death	Ferroptosis-related signaling; mitochondrial dysfunction; oxidative lipid damage	Mainly *in vitro* oxidative stress models	More stable than H_2_O_2_ and useful for modelling sustained oxidative injury	Can cause marked cytotoxicity at high concentrations, limiting physiological relevance	Yan et al. (2024), Alhadi et al. (2025)
H/R	Mimics ischemia-reperfusion injury, producing a burst of reactive oxygen species during reoxygenation and inducing mitochondrial damage	HIF-1α signaling; mitochondrial apoptotic pathways; ischemia-reperfusion stress responses	Both *in vitro* and *in vivo* models	Clinically relevant for modelling ischemic and vascular components of glaucoma	More complex to standardize; reactive oxygen species induction is less direct and less controlled	Chidlow et al. (2017), Jassim et al. (2021)
Glutamate excitotoxicity	Excess extracellular glutamate induces Ca^2+^ influx, mitochondrial dysfunction, and secondary reactive oxygen species production, contributing to RGC injury	Ca^2+^-dependent apoptotic signaling; MAPK/ERK activation; mitochondrial oxidative stress	Mainly *in vitro* RGC models	Highly relevant to RGC neurodegeneration	Not a pure oxidative stress model because excitotoxic and oxidative mechanisms occur together	Maher and Hanneken (2005)
TNF-α	Activates inflammatory signaling, leading to secondary reactive oxygen species generation through NADPH oxidase activation and mitochondrial dysfunction	NF-κB signaling; NADPH oxidase activation; inflammatory oxidative stress; mitochondrial dysfunction	Mainly *in vitro* models; also relevant to *in vivo* inflammatory glaucoma mechanisms	Captures the inflammatory oxidative crosstalk observed in glaucoma	Indirect inducer of oxidative stress and therefore less suitable as a primary reactive oxygen species model	Cueva Vargas et al. (2015)

Abbreviations: H_2_O_2,_ hydrogen peroxide; tBHP, tert-butyl hydroperoxide; H/R, hypoxia/reoxygenation; ROS, reactive oxygen species; NF-κB, nuclear factor kappa B; MAPK, mitogen-activated protein kinase; ERK, extracellular signal-regulated kinase; HIF-1α, hypoxia-inducible factor 1-alpha; NADPH oxidase, nicotinamide adenine dinucleotide phosphate oxidase; TNF-α, tumor necrosis factor alpha; TM, trabecular meshwork; RGC, retinal ganglion cell.

### 2.2 Trabecular meshwork cells

In TM-specific systems, oxidative stress is often induced with H_2_O_2_, tBHP, and TNF-α, by applying mechanical strain or shear stress to mimic elevated IOP, or by inducing mitochondrial dysfunction to increase intracellular ROS (Saccà and Izzotti, 2014; Vernazza et al., 2019; Bikuna-Izagirre et al., 2022). These tools allow researchers to study and investigate oxidative stress pathways implicated in TM dysfunction in glaucoma. In response to oxidative stress, TM cells exhibit several pathological changes. Structural alterations include disruption of the actin cytoskeleton and changes in cell junctions leading to reduced cellular integrity. TM cells exhibit accelerated cellular senescence reflected by increased β-galactosidase activity and elevated p16 expression. Oxidative stress also induces inflammatory activation, such as increased secretion of interleukins, including interleukin-6 (IL-6) and interleukin-1β (IL-1β), and activation of nuclear factor kappa B (NF-κB) signaling, resulting in tissue damage. Mitochondrial failure is characterized by decreasing adenosine triphosphate (ATP) levels, dysregulated membrane potential, and poor cellular energy production (Tezel, 2004; Vernazza et al., 2019). Also, remodeling of the ECM occurs with overproduction of fibronectin and collagen, which contributes to the increased resistance in the AH outflow pathway that is a major determinant of glaucoma progression (Saccà and Izzotti, 2014; Vernazza et al., 2019).

Several different biomarkers and tests have been developed to assess oxidative damage in TM cells and are meant to detect specific molecular alterations. 2′,7′-dichlorodihydrofluorescein diacetate (DCFDA) fluorescence imaging is utilized to measure global ROS burden in living cells (Eruslanov and Kusmartsev, 2010). MitoSOX Red is often used to detect mitochondrial superoxide (Robinson et al., 2006). Profiling of antioxidant enzymes analyzes variations in activity of superoxide dismutase (SOD), catalase, and glutathione peroxidase (GPx) and provides information on cell defenses against oxidative stress (Saccà and Izzotti, 2014; Weydert and Cullen, 2010).

Recently, primary TM cultures in 2D were compared with 3D cultures in different oxidative circumstances. 3D culture systems allow TM cells to proliferate in a native tissue-like manner. The TM cells grown in 3D exhibit increased modulation of ECM, resistance to ROS, and apoptotic resistance (Vernazza et al., 2019). These data suggest that 3D models can offer a more physiologically relevant platform to study glaucoma TM failure. TM cell models are therefore useful to research the effect of oxidative stress on AH outflow and IOP. Primary TM cultures are still the most popular option, but iPSC-derived TM-like cells have been produced as a human-specific alternative. While primary TM cultures are more commonly used, iPSC-derived TM cells can also replicate TM-specific stress responses such as accelerated extracellular matrix turnover and molecular alterations (Abu-Hassan et al., 2015; Zhu et al., 2020; Sui et al., 2021). Because iPSC-derived TM-like cells can be generated from donor-specific genetic backgrounds and can reproduce several TM-like functional properties, they may provide a useful platform for studying patient-specific TM responses, AH outflow regulation, and potential IOP-modulating strategies (Abu-Hassan et al., 2015; Zhu et al., 2020; Sui et al., 2021).

### 2.3 Human RGCs and RGC-surrogate models

In TM cells, oxidative stress mainly leads to dysregulation of IOP as an upstream pathogenic event. RGCs are the major downstream neuronal target of ROS-induced damage in glaucoma. RGCs are the main neurons that transmit visual information from the retina to the brain (Nickells et al., 2012; Aires, Ambrósio, and Santiago, 2017).

RGC cultures provide a direct platform to investigate oxidative stress-related neuronal degeneration and test neuroprotective treatments Kang et al. (2021). The restricted availability and survival of human RGCs represent a major problem, and alternate *in vitro* models are explored (Sluch et al., 2015; Rohowetz et al., 2018). RGC-5 cells were historically used as an RGC-like model, but they are now considered not relevant because they were later shown to be of mouse origin and phenotypically similar to 661W photoreceptor-like cells; therefore, findings from this model should be interpreted cautiously and not treated as direct evidence from human RGCs (Van Bergen et al., 2009; Krishnamoorthy et al., 2013). In contrast, primary rodent RGCs obtained by immunological panning utilizing markers such as Thy1.1 or Brn3a are technically challenging but have greater physiological value. Also, SH-SY5Y neuroblastoma cells are employed as surrogates for neurons; however, they are not retina-specific (Rohowetz et al., 2018). Together these models offer complementary systems to explore RGC degeneration with a trade-off between experimental feasibility and biological relevance (Sluch et al., 2015; Aires et al., 2017; Rohowetz et al., 2018; Hayes et al., 2026). Oxidative stress induces pathogenic alterations in RGCs that are similar to the features of glaucomatous optic neuropathy.

Caspase activation, release of cytochrome c from mitochondria, and the changed balance between Bcl-2 and Bax proteins are all involved in the initiation of neuronal apoptosis and programmed cell death. Mitochondrial fragmentation is also seen, an early marker of cell death due to disruption of mitochondrial integrity and function (Tezel, 2004; Nickells et al., 2012). Oxidative stress downregulates synaptic markers in RGCs such as PSD-95 and synaptophysin, suggesting early synaptic impairment before apoptosis (Williams et al., 2013; Rohowetz, Kraus, and Koulen, 2018).

Oxidative stress also influences neurotrophic signaling pathways crucial for RGC survival, such as BDNF/TrkB signaling. Glaucomatous susceptibility includes such impairment, and BDNF signaling recovery promotes RGC function and structure in experimental models (Domenici et al., 2014; Gupta et al., 2014; Kimura et al., 2016; Wójcik-Gryciuk et al., 2020). Oxidative damage also impacts the cytoskeletal components, causing axonal transport disturbances, which makes it difficult for essential materials to move along the axons of RGCs, leading to deterioration of neuronal function (Nuschke et al., 2015; Dias et al., 2022). Another key consequence is dysregulated autophagy and mitophagy, leading to abnormal clearance and accumulation of damaged organelles, a rise in the production of ROS, and a feed-forward cycle of cell deterioration (Maddineni et al., 2026; Garza-Lombo et al., 2020; Stavropoulos et al., 2023). These findings indicate that dysregulated autophagy is a stress response and could serve as a promising therapeutic target (Lin and Kuang, 2014; Ju et al., 2023). Together, these pathological changes reproduce key aspects of glaucomatous optic neuropathy and support the use of RGC models for testing neuroprotective strategies (Johnson et al., 2011; Wang et al., 2014; Mallone et al., 2020; Ma et al., 2025).

Human iPSC-derived RGCs have emerged as a novel approach to rodent-derived and immortalized RGC models, as they capture patient-specific genetic backgrounds and human-specific oxidative stress responses. iPSC-derived RGCs provide a human-relevant system with RGC-like transcriptional and morphological characteristics and susceptibility to oxidative stress, particularly in disease-associated genetic backgrounds. The iPSC-derived RGCs express important markers such as Brn3b and RBPMS and are sensitive to ER stress and mitochondrial oxidative damage (Sluch et al., 2015; Rabesandratana et al., 2020; Abd Alhadi et al., 2025).

iPSC-derived RGCs/ROs from patients with glaucoma show increased ROS, mitochondrial dysfunction, and increased apoptotic signaling, suggesting a correlation with disease genotypes and oxidative sensitivity (Gomes et al., 2022; Lei et al., 2024; Verma et al., 2024). In summary, the optimal choice of RGC model is a balance between biological relevance and practicality, and iPSC-derived RGCs provide an outstandingly relevant model for human oxidative stress responses. iPSC-derived models are particularly valuable for studying the genotype-dependent susceptibility to oxidative stress and for modeling glaucoma associated with certain genetic risk variants (Sluch et al., 2015; Rabesandratana et al., 2020).

In addition, these models permit thorough studies of the role of oxidative stress in early synaptic degeneration and trophic deprivation in glaucoma (Williams et al., 2013; Gupta et al., 2014; Nuschke et al., 2015).

### 2.4 Retinal organoids

Retinal organoids (ROs) may recapitulate the layered structure of the human retina and include endogenously differentiating RGC populations (Capowski et al., 2019; O’Hara-Wright and Gonzalez-Cordero, 2020). This is further confirmed by organoid investigations that show strong RGC specification, neurite extension, and susceptibility to neurodegenerative stresses (Fligor et al., 2021). These properties make organoids useful for studying oxidative stress in a tissue-like environment (Hayes et al., 2026). They also reflect disease-related changes, including reduced inner plexiform layer synaptic density (Capowski et al., 2019; O’Hara-Wright and Gonzalez-Cordero, 2020). Human induced pluripotent stem cell (hiPSC)-derived ROs are complex 3D culture models that imitate retinal architecture but lack vascular and immunological components and complete maturity. Progressive development of these organoids can create stratified retinal layers, including RGCs, photoreceptors, bipolar cells, and Müller glia.

ROs are being used to study RGCs’ specific susceptibility to oxidative stress and mitochondrial dysfunction, two hallmarks of glaucomatous neurodegeneration (O’Hara-Wright and Gonzalez-Cordero, 2020). The glaucoma-associated Optineurin (*OPTN*-*E50K*) mutation in RO-derived RGCs causes cell-intrinsic neurodegenerative phenotypes, including impaired neurite integrity and functional deficits, suggesting that disease-causing genetic variants may make RGCs vulnerable in 3D retinal environments (VanderWall et al., 2020). RGC mitochondrial and degenerative characteristics are replicated in iPSC-derived glaucoma models. Mechanistic studies of RGC formation and vulnerability can be done in novel organoid models with glaucoma-related genetic variants such as *ATOH7* disruptions (Correia et al., 2025). Organoids from donors with *OPA1* mutations show poor RGC development and mitochondrial dysfunction, validating disease-specific genetic models for oxidative vulnerability thresholds (Lei et al., 2024). ROs fall between simple cell cultures and *in vivo* systems (Manafi et al., 2021; Chakrabarty et al., 2024; Ma, Daniszewski, and Pébay, 2025).

ROs, like iPSC-derived models, allow researchers to analyze disease processes in the context of individual genetic backgrounds, especially for glaucoma variations with genetic anomalies. These models enable high-throughput pharmacological screening of antioxidant and neuroprotective agents to reduce oxidative stress-induced damage (Rabesandratana et al., 2020). ROs and iPSC-derived retinal systems maintain the human biological context, making them suitable for studying how oxidative stress regulates trophic signaling networks and synaptic maintenance pathways, which are promising neuroprotective targets for glaucoma (Kimura et al., 2016; Mallone et al., 2020).

iPSC-derived ROs also support longitudinal assessment of retinal development and disease-related phenotypes in a human genetic context, making them useful for modeling genotype-specific oxidative vulnerability and testing therapeutic strategies (Lei et al., 2024; Verma et al., 2024; Theodore et al., 2025).

By overcoming current limitations and leveraging their unique capabilities, iPSC-based models hold immense potential to advance our understanding of glaucoma and accelerate the discovery of novel treatments. These systems are particularly powerful for isolating ROS-sensitive phenotypes and genotype-specific vulnerabilities; however, their findings should still be interpreted within the broader context of tissue-level and systemic oxidative stress (Verma et al., 2024; Ma et al., 2025).

Thus, ROs provide a model that sits between simple cell cultures and *in vivo* systems that can be useful for studying multicellular responses to oxidative stress and exploring possible genetic risk factors in a human context (Manafi et al., 2021; Chakrabarty et al., 2024; Ma, Daniszewski and Pébay, 2025).

### 2.5 Organ-on-chip and advanced co-culture platforms

Emerging co-culture and organ-on-chip technologies are promising complementary platforms for oxidative stress investigations related to glaucoma. These models can enable controlled fluid flow, oxygen gradients, tissue compartmentalization, and interactions among retinal, TM, glial, vascular, and immune cells. Thus, these models can serve as a bridge between simple 2D cultures and *in vivo* models. However, the application of these models in glaucoma research is still limited by technical complexity, lack of full standardization, and difficulties in mimicking chronic IOP-induced optic nerve injury (Gao et al., 2025; Lu et al., 2025).

## 3 *In vivo* models

Glaucoma models can be categorized into genetic models and secondary inducible models. Secondary inducible models may be further divided into IOP-dependent and IOP-independent models depending on their underlying mechanism. *In vivo* models are needed to examine the combination of oxidative stress with elevated or normal IOP, neuroinflammation, and vascular dysregulation in a physiologically integrated setting.

Genetic models can be divided further into IOP-dependent models such as DBA/2J and *MYOC*-associated models, and IOP-independent or normal-tension glaucoma models such as *OPTN, TBK1, SLC1A3*/EAAT1/*GLAST, WDR36,* and *SOD1*-deficient models. This classification is significant as oxidative stress may be the consequence of several upstream triggers, such as compromised AH outflow, mitochondrial dysfunction, deficient autophagy or mitophagy, excitotoxicity, and reduced antioxidant defenses.

### 3.1 Rodent models (mouse, rat)

Rodents are the most widely used experimental species in glaucoma studies because of their genetic tractability, comparatively low cost, and well-defined ocular anatomy (Bugara et al., 2024; Di Marsico et al., 2025). Rodent eyes are smaller than human eyes but have comparable critical anatomical characteristics, including TM and a lamina cribrosa-like area (Tamm et al., 2017).

#### 3.1.1 Inducible ocular hypertension models

##### 3.1.1.1 Microbead occlusion model

Glaucoma studies often use the microbead occlusion model. Polystyrene or magnetic microbeads introduced into the anterior chamber of the eye block TM outflow through the AH (Sappington et al., 2010; Fard et al., 2025; Mead, 2023). This mechanical blockage impairs AH outflow, leading to a sustained elevation in IOP and the subsequent induction of oxidative stress within the retina and optic nerve head (ONH) (Fard et al., 2025). Oxidative stress in this model is associated with impaired tissue perfusion and vascular dysregulation, providing an environment conducive to downstream mitochondrial dysfunction and inflammatory activation (Samsel et al., 2011; Bunker et al., 2015; Karg et al., 2023).

In this model, ROS levels are usually evaluated by staining retinal tissue sections with dihydroethidium (DHE), which allows the visualization of superoxide radicals (Jassim and Inman, 2019). The higher the fluorescence intensity, the greater the accumulation of ROS in the tissues, and this correlates well with the duration and magnitude of IOP elevation (Fan Gaskin et al., 2021). Protein carbonyl tests are also used to measure oxidative damage to proteins, giving more evidence of oxidative load in damaged tissues. Oxidative damage in rat models may potentially activate pathways associated with ferroptosis, a recently identified cause of RGC death (Yao et al., 2023).

Pathological outcomes of this paradigm include RGC apoptosis (programmed cell death), axonal degeneration within the ON, and thinning of the RNFL, thereby recapitulating major aspects of human glaucoma (Huang et al., 2018). Such changes are often accompanied by increased levels of markers of lipid peroxidation such as malondialdehyde (MDA), showing oxidative damage to lipid membranes, and suggesting that ROS exacerbate glaucomatous neurodegeneration (Moreno et al., 2004). The capacity of this model to reproduce selected IOP-induced oxidative stress and structural damage makes it useful for investigating pressure-related retinal injury and evaluating treatments that may reduce oxidative harm (Fard et al., 2025). This model is suited for evaluating acute IOP-triggered oxidative stress and early neurodegenerative alterations, but it does not mimic the chronic development of human glaucoma. The microbead occlusion model is associated with a rapid IOP increase, often within hours to days after injection, which may induce acute ischemic damage pathways apart from the chronic, progressive oxidative stress in primary open-angle glaucoma (POAG). This restriction should be taken into account when extending the ROS-related processes found in this model to human disease.

Advantages include surgical repeatability and control of IOP elevation with measurable outcomes like flat-mount Brn3a + RGC counts, optical coherence tomography (OCT)-based RNFL, and pattern electroretinography (pERG). POAG is difficult to extrapolate directly, as there is no biological damage to the TM, no systemic metabolic comorbidities, and the IOP variability is strain-dependent (Sappington et al., 2010; Morgan and Tribble, 2015).

##### 3.1.1.2 Episcleral vein cauterization (EVC)

EVC is a well-established glaucoma model in which selected episcleral veins are thermally sealed to impair AH outflow, resulting in sustained IOP elevation. Compared with the microbead occlusion model, EVC generally produces a more chronic pressure-elevation paradigm and is useful for studying progressive RGC injury and ON degeneration (Shareef et al., 1995; Ruiz-Ederra and Verkman, 2006). This chronic ocular hypertension causes hypoxia-like conditions within the retina, which contribute to oxidative stress by boosting NADPH oxidase (NOX) activity and enhancing mitochondrial ROS formation, both of which are essential drivers of glaucomatous neurodegeneration. Immunohistochemistry techniques are used to detect specific markers to assess the extent of oxidative injury in this model, including nitrotyrosine, 4-hydroxynonenal (4-HNE), and oxidized DNA bases such as 8-hydroxy-2′-deoxyguanosine (8-OHdG), which provide precise spatial localization of oxidative damage within retinal and ON tissues (Ruiz-Ederra and Verkman, 2006; Duarte, 2021; Fan Gaskin et al., 2021).

EVC-induced continuous oxidative stress triggers a cascade of pathological alterations, including RGC dendritic pruning that compromises their connection, function, and mitochondrial enlargement in ON axons, indicating impaired mitochondrial integrity. Astrocytic gliosis, defined by the reactive proliferation of astrocytes, further exacerbates the neurodegenerative environment and adds to tissue damage. These documented changes are remarkably similar to the progressive neuronal and glial alterations characteristic of human glaucoma, thus making the EVC model a useful tool for investigating the mechanisms of chronic oxidative stress and evaluating potential therapeutic strategies for reducing ROS-mediated damage in glaucoma (Sapienza et al., 2015). EVC outperforms microbead models in mimicking chronic oxidative stress and progressive neurodegeneration, which is valuable for long-term disease mechanism studies.

Importantly, EVC immediately impedes venous outflow, creating an acute ischemic insult that is itself complicated by the following increase in IOP. Thus, oxidative stress reported in this model is not only due to IOP-dependent processes but may in part represent surgical ischemia. This caveat should be taken into account when evaluating ROS-driven results in EVC-based glaucoma investigations. Key advantages include sustained IOP elevation suited for chronic oxidative stress profiling, with endpoints such as ON axon counts and nitrotyrosine/4-HNE immunohistochemistry (Fan Gaskin et al., 2021). Key limitations include the confounded surgical ischemic insult, high inter-animal IOP variability, and larger cohort size requirements for adequate statistical power.

##### 3.1.1.3 Silicone oil-induced ocular hypertension model

In this model, silicone oil is injected into the anterior chamber, where it obstructs AH outflow through the iridocorneal angle and trabecular pathway, resulting in sustained IOP elevation. Its inducibility and reversibility allow researchers to discriminate pressure-dependent insult from pressure-lowering recovery responses by quickly regulating IOP following silicone oil removal. In the study conducted by Zhang et al. (2019), the model showed elevated IOP, loss of RGCs, degeneration of ON axons, and impaired visual function.

The model is useful for the study of chronic glaucomatous stress as it minimizes variability in the bead model and allows controlled induction of ocular pressure. In the absence of ROS, lipid peroxidation, mitochondrial markers, or antioxidant enzyme assays, oxidative stress is often inferred from downstream mitochondrial injury, neuroinflammation, RGC loss, or antioxidant-response measures.

##### 3.1.1.4 Hypertonic saline model

In this model, hypertonic saline is injected into the episcleral veins to scar/sclerose aqueous outflow. This leads to sustained IOP elevation and ON damage in rats. One group (Tezel et al., 2005) used this paradigm to highlight oxidative protein alterations and stress-related immune/glial responses in experimental glaucoma. Thus, this model is useful for investigating pressure-related oxidative protein injury, although the inflammatory response caused by episcleral vein manipulation should be considered when interpreting ROS-related findings.

##### 3.1.1.5 Hyaluronic acid/sodium hyaluronate anterior chamber model

Repeated injections of hyaluronic acid into the anterior chamber restrict outflow and increase IOP. One study (Moreno et al., 2004) showed that retinal antioxidant defenses, particularly the activities of superoxide dismutase and catalase, were reduced, indicating that oxidative stress in the retina might be involved in glaucomatous cell death. This may simulate pressure-driven retinal oxidant imbalance, but repeated injections may result in inflammation or anterior segment damage. Therefore, this model may be most suitable for studying IOP-associated antioxidant depletion, while it is less ideal for isolating oxidative stress from injection-related inflammatory effects.

##### 3.1.1.6 Laser-induced ocular hypertension model

Laser photocoagulation of TM or episcleral/limbal veins decreases aqueous outflow and increases IOP. It replicates ocular hypertension, RGC loss, and ON injury in rats and mice. In this paradigm, oxidative stress is suggested by pressure-induced mitochondrial failure, ischemia-like stress, and glial activation; however, direct evidence is inconsistent amongst studies (Grozdanic et al., 2003; Yun et al., 2014).

#### 3.1.2 Genetic models

Genetic models provide crucial insight into both IOP-dependent and IOP-independent causes of illness. The DBA/2J animal (D2) with *Tyrp1* and *Gpnmb* mutations has iris atrophy, reduced outflow of AH, and subsequent elevated IOP. Instead, models linked to *TBK1* are indicative of normal-tension glaucoma and show neurodegenerative processes independent of IOP. *GLAST*-deficient mice also exhibit RGC degeneration via excitotoxicity and oxidative stress without IOP increase.

These genetic models also vary in their oxidative stress profiles, with *GLAST* deficiency increasing excitotoxic production of reactive oxygen species, and DBA/2J animals presenting with progressive oxidative damage linked to persistent IOP increase and neuroinflammation.

##### 3.1.2.1 DBA/2J mice

In the inbred DBA/2J mice, mutations in the *Tyrp1* and *Gpnmb* genes are responsible for glaucoma (Anderson et al., 2002; Bugara et al., 2024; Shim et al., 2026).

DBA/2J mice are often utilized in chronic, progressive glaucoma studies because of their spontaneous disease model. Such mutations induce a chronic metabolic load on the retina and ONH with ROS accumulation and neuroinflammation (Thompson et al., 2025; Shim et al., 2026). Age-dependent RGC death, ON cupping, and TM extracellular matrix remodeling produce IOP elevation and neurodegeneration comparable to human POAG. In this approach, the quantity of ROS is used to quantify the oxidative damage using DHE, MitoSOX label, and Western blot analysis (Di Marsico et al., 2025).

Other genetic models, such as those that overexpress NOX enzymes or turn out antioxidant enzymes like SOD in mice, enhance ROS production or weaken antioxidant defenses (Fan Gaskin et al., 2021). These genetic changes exacerbate glaucomatous neurodegeneration, highlight oxidative stress, and provide a solid foundation for ROS-reducing therapy trials. Although rabbits and non-human primates (NHPs) have ocular anatomy and AH dynamics similar to those of rats, they are more useful in translational glaucoma studies. To study age-dependent oxidative stress and neuroinflammatory interplay in glaucoma development, this paradigm is useful (Howell et al., 2007; Porciatti et al., 2007).

There are several relevant outcomes, including serial intraocular pressure measurements, flat-mounted RGC soma density, grading of ON damage (0–4 scale), and OCT-derived RNFL thickness. The translational extrapolations are limited by the high inter-litter variability in IOP (at least 15 mice per group) and the *Tyrp1/Gpnmb* pigmentary mechanism, which is distinct from the pathophysiology of POAG TM in humans (Turner et al., 2017).

##### 3.1.2.2 OPTN (optineurin) models

OPTN mutations, especially E50K, have been linked to normal-tension glaucoma and progressive RGC degeneration. OPTN normally functions in selective autophagy and mitophagy to mediate the clearance of dysfunctional mitochondria. E50K mutation impairs this mechanism and is associated with mitochondrial fragmentation, faulty mitochondrial clearance, increased ROS generation, defective autophagic flux, and activation of apoptotic pathways in RGCs. These findings suggest that mitochondrial dysfunction and impaired autophagy are important mechanisms of OPTN-associated glaucomatous neurodegeneration (Shim et al., 2016; Zhang et al., 2021).

##### 3.1.2.3 TANK-binding kinase 1 (*TBK1*) model

TBK1 duplications are a genetic cause of normal-tension glaucoma is TBK1 duplications. OPTN phosphorylation by TBK1 regulates selective autophagy, mitophagy, and innate immunity. TBK1 activity alters mitochondrial quality control, accumulates faulty mitochondria, and increases oxidative stress signaling. Molecular studies indicate that dysregulation of OPTN-TBK1, NF-κB, and autophagy pathways decreases cellular homeostasis and increases RGC degeneration risk. Neuroprotective medicines targeting mitochondrial quality control and autophagy signaling are suggested by these models (Tucker et al., 2014; Fingert et al., 2016).

##### 3.1.2.4 *MYOC* (myocilin) models

Hereditary POAG is usually caused by *MYOC* mutations like Y437H and G364V. In TM cells, myocilin misfolding triggers the UPR via PERK, ATF6, IRE1α, CHOP, and GRP78/BiP signaling in the ER. ER stress degrades TM and impairs aqueous fluid outflow by causing mitochondrial failure, ROS generation, oxidative protein degradation, and cell death. Therapeutic studies demonstrated that 4-phenylbutyrate (4-PBA) reduces ER stress, enhances myocilin, lowers IOP, and preserves retinal structure (Joe et al., 2003; Zode et al., 2011; 2012).

##### 3.1.2.5 WD repeat domain 36 (WDR36) models

WDR36 supports nucleolar homeostasis by processing ribosomal RNA. Experimental results reveal that *WDR36* deletion activates p53-dependent stress pathways, inhibits ribosome biogenesis, and increases oxidative damage sensitivity. *WDR36* disruption changes *BAX*, p53, and mitochondrial stress regulator expression, reducing stress adaptation and increasing ROS-mediated damage sensitivity. Even without knowing *WDR36*’s pathogenic role, our findings imply that oxidative stress and mitochondrial malfunction cause glaucoma-associated neurodegeneration (Skarie and Link, 2008; Chi et al., 2010; Meer et al., 2021).

##### 3.1.2.6 EAAT1/*SLC1A3* (*GLAST*) knockout models

The *GLAST*-deficient mouse model of normal-tension glaucoma is well-known. GLAST is a glutamate transporter expressed by Muller glia and contributes to retinal glutamate homeostasis and glutathione synthesis. GLAST loss induces extracellular glutamate accumulation, NMDA receptor-mediated excitotoxicity, glutathione depletion, mitochondrial dysfunction, and excessive ROS production. RGC degeneration is caused by apoptosis despite normal IOP. These findings suggest oxidant and glutamate-modulating treatments for glaucoma-like neurodegeneration caused by oxidative stress and excitotoxicity alone (Harada et al., 2007; 2010).

##### 3.1.2.7 *SOD1*-deficient models

Deficiency of superoxide dismutase 1 (*SOD1*) relates oxidative stress to retinal neurodegeneration. *SOD1* catalyzes the dismutation of superoxide radicals into hydrogen peroxide and oxygen; therefore, its loss causes ROS, oxidative DNA damage, lipid peroxidation, and mitochondrial dysfunction. In *SOD1*-deficient animals, age-dependent retinal degeneration and RGC loss are associated with elevated oxidative damage indicators, supporting the idea that defective antioxidant defenses cause glaucomatous optic neuropathy. These models regularly evaluate antioxidant and mitochondrial-targeted neuroprotective therapies (Yuki et al., 2011).

#### 3.1.3 Acute axonal injury model: optic nerve crush (ONC)

ONC is not an ocular hypertension model because it directly injures the ON without increasing IOP or impairing AH outflow. However, it is widely used to study synchronized RGC axonal degeneration, apoptosis, mitochondrial and metabolic dysfunction, oxidative stress, and neuroprotective responses. Therefore, ONC is best described as an optic neuropathy or RGC injury model that captures the axonal injury and neurodegenerative components relevant to glaucoma, but not the pressure-dependent or outflow-dependent mechanisms of ocular hypertension. Studies using ONC have shown retinal and ON gene-expression changes, caspase-mediated apoptosis, altered energy metabolism, mitochondrial injury, and SIRT1-associated RGC neuroprotection (Tang et al., 2011; Sharma et al., 2014; Choudhury et al., 2015; Daniel et al., 2018; Zhu et al., 2020; Tsuji et al., 2023).

ONC is particularly useful for studying RGC survival, apoptosis, axonal degeneration, and neuroprotective responses after direct optic nerve injury. Previous ONC studies have reported retinal and ON gene-expression changes linked to neurodegenerative pathways, caspase-mediated apoptosis, altered energy metabolism, and mitochondrial dysfunction (Tang et al., 2011; Sharma et al., 2014; Choudhury et al., 2015; Daniel et al., 2018; Zhu et al., 2020; Tsuji et al., 2023). However, because ONC bypasses IOP elevation and AH outflow impairment, findings from this model should be interpreted as evidence of RGC/ON mechanisms rather than pressure-dependent glaucoma pathology.

### 3.2 Rabbit models

Rabbit models are suitable for studying surgically and pharmacologically induced glaucoma due to their large anterior chambers and ease of drug administration (Bouhenni et al., 2012). Rabbits could serve a good model to simulate neurodegenerative processes in glaucoma because they show similarities with humans in the ON, lamina cribrosa, and astrocyte architecture (Perlman, 2009). Laser photocoagulation of the TM or circumferential cryogenic damage may increase IOP (Johnson and Tomarev, 2010; Bouhenni et al., 2012). Rabbit aqueous humor is biochemically comparable to human aqueous humor (Edward and Bouhenni, 2011). Rabbits are validated experimental models for oxidative-stress studies, as paraquat-induced retinal oxidative injury causes mitochondrial dysfunction, ROS elevation, and photoreceptor degeneration (Kanan et al., 2025). Evidence of oxidative stress in rabbit glaucoma models includes increased malondialdehyde and nitric oxide and diminished antioxidant enzymes (SOD, catalase, and GPx) in chronic ocular hypertension rabbits (Panchal et al., 2017). The present results indicate that rabbit models might be beneficial for the study of IOP-induced oxidative damage and antioxidant-based neuroprotection. Sustained IOP elevation may induce ischemia-reperfusion-like injury in retinal and ON tissues, leading to increased ROS production (Hu et al., 2025). There have been recent studies identifying oxidative stress mechanisms in rabbit glaucoma. Rabbits have a thinner sclera, more choroidal blood flow, and a larger lens than humans, which may make assessment of the posterior segment difficult or challenging to practically investigate (Werner et al., 2006).

### 3.3 Non-human primate models

Rhesus and cynomolgus monkeys have ON heads and lamina cribrosa that are comparable to those of humans and are considered the gold standard for preclinical glaucoma study (Evangelho et al., 2019; Di Marsico et al., 2025). The anterior TM, the arrangement of lamina cribrosa collagen fibers, the flat shape of the optic disc, and the amount of RGCs are similar (Yan et al., 2015). Glaucoma is induced in non-human primates by raising IOP via laser trabeculoplasty or episcleral vein sclerosis.

Few direct oxidative stress measurements have been made in non-human primate glaucoma models. Theoretically, oxidative stress indicators are translatable from rodent and human studies to the NHP study (Evangelho et al., 2019). NHP are neuroanatomically comparable to humans and hence may be used to explore the reactions of central visual pathways to glaucoma, including alterations in higher-order visual centers (Almasieh and Levin, 2017; Burgoyne, 2015) proposed *in vivo* validation of non-invasive structural and functional tests for early and chronic glaucomatous development. These models show optic disc cupping, peripapillary atrophy, and selective susceptibility of RGC axons as in the genuine glaucoma pathogenesis (Fortune et al., 2016). However, the use of NHPs in glaucoma studies is limited by a lack of genetic resources, transgenic lines, ethical considerations, high prices, and specialized housing and handling facilities (Chen et al., 2016; Almasieh and Levin, 2017).

Despite these constraints, NHP models uniquely support clinical-grade endpoints, including Humphrey visual field testing, Heidelberg retina tomography (HRT) optic disc cupping, macular ganglion cell complex (GCC), OCT, and multifocal ERG, that directly parallel human glaucoma trial outcome measures and are unavailable in rodent systems (Harwerth et al., 2002; He et al., 2014; Burgoyne, 2015; Wilsey et al., 2016).

### 3.4 Other experimental species models

Although mammalian models are widely used in glaucoma research, other non-mammalian models such as zebrafish and *Drosophila* have proven to be highly efficient alternatives. These models are valuable because they allow rapid genetic manipulation, high-throughput screening, and assessment of stress-related cell death in ocular tissues. Zebrafish share considerable genetic and structural similarity with humans, including several conserved features of eye anatomy. Moreover, they reproduce rapidly and are transparent during the early stages of development, making it easier to observe and study the development and structural changes of the eye and the surrounding tissue without any invasive procedures. Disruption of the *FOXC1* transcription factor and its downstream target *FOXO1A* was shown to impair cellular resistance to oxidative stress and increase cell death in both TM cells and the developing zebrafish eye (Berry et al., 2008). Zebrafish models, including *SIX6*, *FOXC1*, and *bugeye* mutants, as well as chemical and oxidative stress models, have also been used to study glaucoma-associated pathways and RGC neurodegeneration (Hong and Luo, 2021).

Similarly, *Drosophila* offers fast and high-throughput genetic screening owing to the complex compound eye of the organism. *Drosophila* may be useful for testing candidate glaucoma genes identified from human genome-wide association studies (GWAS), in terms of when screening conserved pathways involved in neuronal stress, degeneration, or survival. Overexpression of human *TIGR/MYOC* in the *Drosophila* eye was shown to cause structural eye defects and alter the expression of glaucoma-associated transcripts, including changes in the neurodegeneration-related protein Swiss Cheese and its human ortholog, supporting the use of fruit flies as a screening model for glaucoma-related genes and stress-response pathways (Borrás et al., 2003).

## 4 Clinical samples

ROS are short-lived and respond quickly; therefore, assessing them in human ocular tissues and biological fluids is difficult. Oxidative stress markers have been associated with glaucoma pathology in surgical biopsies, AH, vitreous humor (VH), plasma, and peripheral blood mononuclear cells (PBMCs) in the eyes of donors. Genetic evidence from blood-based GWAS also points to the involvement of oxidative stress-related pathways in glaucoma susceptibility. Several glaucoma risk loci are located near genes with known roles in redox regulation. *TXNRD2* encodes a mitochondrial antioxidant enzyme, *OPTN* regulates selective mitophagy and its E50K mutation impairs mitochondrial quality control, *CDKN2B-AS1* is linked to RGC senescence, and *ABCA1* influences membrane lipid composition and redox signaling. Additional loci, including *SIX6* and *FNDC3B*, further support genetic contributions to ON/RNFL structural vulnerability and glaucoma susceptibility (Ulmer Carnes et al., 2014; Wiggs and Pasquale, 2017; Shiga et al., 2018; Verma et al., 2024).

### 4.1 Post-mortem samples

Human eyes provide the optimal opportunity for assessing oxidative damage in *postmortem* glaucoma-afflicted tissues. ROS-mediated stress is substantially accumulated in the TM, retina, and ONH of glaucoma donors (Patel et al., 2020; Monavarfeshani et al., 2023; Li et al., 2026). TM tissue from the juxtacanalicular region shows increased levels of 8-OHdG, 4-HNE, and protein carbonyl adducts indicative of oxidative damage to DNA, lipids, and proteins (Izzotti et al., 2003; Saccà et al., 2005). Several studies have shown elevated NOX1/NOX4 expression in the retina and ONH. RGCs show mitochondrial DNA deletions, oxidized phospholipids, and elevated oxidative stress. Ultrastructural studies have shown that hallmarks of oxidative mitochondrial injury include swelling, cristae rarefaction, and vacuolization (Cakir et al., 2023). ONH astrocytes exhibit glial activation, increased nitrotyrosine deposition, and reduced expression of antioxidant enzymes consistent with the concept that sustained ROS exposure leads to axonal degeneration and neuroinflammation in glaucomatous optic neuropathy (Tezel, 2004). Post-mortem tissue collection delay and ischemia may independently increase the amounts of ROS and thus complicate the assessment of oxidative stress indicators in post-mortem tissues. These variables may lead to oxidative damage and need to be taken into account when comparing donor tissue to *in vivo* disease processes.

### 4.2 Surgical biopsies

TM and scleral tissue obtained from trabeculectomy can be assayed for oxidative stress signatures similar to human AH and glaucoma blood biomarkers by Western blotting, RT-PCR for NOX isoforms, and liquid chromatography–mass spectrometry (LC-MS)-based oxidized lipid profiling (Izzotti et al., 2010; Hondur et al., 2017; Gowtham et al., 2025).

In human biospecimens such as AH (Ferreira et al., 2004), VH (Schwab et al., 2020), plasma (Golpour et al., 2025), serum (Shao et al., 2025), and PBMCs (Mohanty et al., 2022), oxidative stress markers may be detected in a minimally invasive manner. AH assays show lower antioxidant capacity and higher MDA, SOD, and GPx activity, protein carbonyls, and advanced glycation end products (AGEs). Vitreous samples reveal elevated protein oxidation, including oxidized albumin, in POAG eyes. Ferreira et al. (2004); Hondur et al. (2017); and Dammak et al. (2023) showed that plasma metabolomics revealed enrichment of oxidative stress-related pathways and reduced mitochondrial respiration in PBMCs of glaucoma patients.

The most consistent oxidative stress findings that are replicated in both experimental and human research are DNA oxidation, lipid peroxidation, protein oxidation, altered antioxidant defenses, mitochondrial dysfunction, and inflammatory or glial activation indicators when tissue allows (Hanyuda et al., 2026). Caution is advised in the interpretation of values derived from blood, serum, plasma, and PBMCs since oxidative stress may be influenced by age, medication, systemic disease, and metabolic comorbidities irrespective of ocular pathology (Baris et al., 2024). Importantly, distinct experimental models represent different aspects of oxidative-stress-related pathogenesis of glaucoma. Studies on controlled ROS signaling and therapy can be conducted using TM cell cultures, RGC models, 3D structures, ROs, and iPSC-derived cells. On the other hand, *in vivo* animal models enable investigation of neuroinflammation, vascular dysregulation, and persistent oxidative stress under high intraocular pressure. To assess oxidative stress markers, multidisciplinary neurodegenerative research has highlighted the need for humanized and translational tissue platforms. Human donor eyes, TM tissue, retinal tissue, and ocular fluids from glaucoma patients all exhibit signs of experimental oxidative damage (Ward et al., 2025). These alternative techniques offer a strong foundation for integrating mechanistic knowledge and clinical significance and underscore the need to choose models that fit the study issue, whether it concerns pharmacologic modulation, genetic predisposition, or disease development. Plasma, serum, or PBMC indicators of oxidative stress may be markers of aging or metabolic comorbidities rather than ocular illness. Their sensitivity to intraocular oxidative stress is fascinating but should be considered cautiously. Together, these data reinforce the significance of oxidative stress biomarkers as markers of disease progression and as possible targets for new neuroprotective therapeutics. Tables 2-4 compare experimental models with pathways of oxidative stress in glaucoma. To make model selection more practical, Table 5 summarizes common research objectives, suitable experimental models, key reasons for selection, and major limitations of each model.

**TABLE 2:** *In vitro* and organoid models of oxidative stress in glaucoma.

Model category	System/model	Common stress inducers	Key pathways	Main outcomes	Key measurable endpoints	Advantages	Limitations	References
*In vitro* (2D)	Trabecular meshwork (TM) cells	H2O2; tBHP; cyclic mechanical stretch; TGF-β2-rich conditions	Mitochondrial dysfunction; NF-κB activation; ER stress; apoptosis; ECM remodeling	TM cell damage; impaired aqueous outflow; increased fibrotic signaling	Intracellular ROS (DCFDA); mitochondrial ROS (MitoSOX); mitochondrial membrane potential (JC-1/TMRE); caspase-3/7; TUNEL; fibronectin/collagen IV/alpha-SMA; TEER or outflow-resistance assays where applicable	Highly controlled exposure conditions; suited for mechanism-first testing of redox signaling and ECM responses; amenable to pharmacologic or gene-perturbation experiments	Lacks 3D outflow architecture, Schlemm’s canal crosstalk, and immune-cell interactions; oxidative dose-response may exceed physiological ranges	Vernazza et al. (2019), Snider et al. (2021), Yan et al. (2024)
*In vitro* (2D)	Retinal ganglion cells (primary RGCs, RGC-5 where historically used, or iPSC-derived RGCs)	H2O2; glutamate excitotoxicity; serum deprivation; mitochondrial toxins	Mitochondrial dysfunction; oxidative stress-induced apoptosis; axonal injury signaling	RGC degeneration; oxidative macromolecular damage	Cell viability (MTT/CCK-8); ROS and mitochondrial ROS; Brn3a/RBPMS-positive RGC counts; neurite/axon length; caspase-3/7; mitochondrial membrane potential; 8-OHdG and lipid peroxidation markers	Cell-specific readout of RGC vulnerability; useful for high-throughput screening of antioxidant or neuroprotective candidates	Limited retinal microenvironment; lacks astrocyte, microglial, and vascular influences that shape oxidative injury in vivo	Maher and Hanneken (2005), Gomes et al. (2022)
*In vitro* (3D)	Retinal organoids	Genetic or disease-associated stress; mitochondrial stress; hypoxic or oxidative challenge	Mitochondrial dysfunction; neurodegenerative pathways; altered synaptic homeostasis	RGC vulnerability; structural and functional deficits	RGC layer thickness; Brn3a/RBPMS staining; ROS burden; ATP or oxygen-consumption rate; apoptosis markers; PSD-95/synaptophysin; calcium imaging or electrophysiology	Better tissue architecture and cell-cell interaction than 2D systems; useful for longitudinal phenotyping of retinal stress responses	Immature retinal state in some protocols; limited vascular, immune, and pressure-loading components	Capowski et al. (2019), Fligor et al. (2021), Lei et al. (2024)
iPSC-derived models	Patient-specific RGCs and outflow-pathway cells	Mutation-associated oxidative stress; mitochondrial dysfunction; proteostasis stress	Mitochondrial dysfunction; neurodegeneration; impaired stress adaptation	Patient-specific disease phenotypes; altered stress resilience	Genotype-phenotype readouts; ROS and mitochondrial ROS; neurite/axon length; apoptosis; OCR/ECAR; transcriptomic or proteomic signatures; response to rescue/drug treatment	High translational relevance; supports modeling of patient-specific susceptibility and treatment response	Costly; variable differentiation efficiency and maturation across lines	Gomes et al. (2022)

**TABLE 3:** Rodent and genetic models of oxidative stress in glaucoma.

Model category	System/model	Common stress inducers	Key pathways	Main outcomes	Key measurable endpoints	Advantages	Limitations	References
*In vivo* (rodent)	Mouse or rat glaucoma models, including DBA/2J (spontaneous genetic), microbead-induced ocular hypertension, Morrison model (episcleral vein hypertonic saline injection), laser photocoagulation, and episcleral vein cauterization	Sustained IOP elevation from aqueous-outflow obstruction; endogenous oxidative stress; NOX-derived ROS; mitochondrial ROS from axonal transport failure; glial inflammatory oxidants	NOX activation; mitochondrial ROS generation; neuroinflammation; axonal transport disruption; apoptotic and glial stress signaling	RGC loss; optic nerve (ON) degeneration; visual dysfunction	IOP time course; Brn3a/RBPMS-positive RGC counts; optic-nerve axon counts; retinal or ONH ROS/NOX expression; Iba1 and GFAP; OCT/ERG/optokinetic readouts	Captures pressure-dependent neurodegeneration in an intact eye; supports longitudinal IOP-structure-function analysis; genetically tractable in mice; includes widely used chronic models such as DBA/2J and Morrison hypertonic-saline injury	Species differences in optic-nerve-head biomechanics; variable IOP elevation across induction methods; some models show procedure-dependent variability and incomplete chronicity; rodent lamina architecture differs from the human lamina cribrosa	( Anderson et al., 2002; Sappington et al., 2010; Dvoriantchikova et al., 2012; Kim et al., 2015; Morrison, Johnson and Cepurna, 2018 )
Genetic models	*TBK1*, *OPTN*, WDR36-associated systems; DBA/2J also provides a spontaneous genetic ocular-hypertension background	Mutant protein misfolding; ER stress; impaired autophagy/mitophagy; mitochondrial dysfunction; redox imbalance	Unfolded protein response; oxidative protein damage; mitochondrial stress; neuroinflammatory signaling	TM dysfunction or RGC vulnerability depending on genotype; chronic neurodegenerative susceptibility	IOP; RGC counts; axon counts; ROS markers; mitochondrial assays; ER-stress markers (BiP, CHOP, XBP1); autophagy markers (LC3, p62); genotype-phenotype correlation	Causally links oxidative injury to defined glaucoma genes; useful for mechanism-driven studies and precision-therapy testing	Single-gene systems may not reproduce the full polygenic and age-related complexity of common POAG; phenotype severity can vary by background strain	( Joe et al., 2003; Chi et al., 2010; Fingert et al., 2016; Shim et al., 2016 )

**TABLE 4:** Large-animal models and human clinical samples in oxidative stress-related glaucoma research.

Model category	System/model	Common stress inducers	Key pathways	Main outcomes	Key measurable endpoints	Advantages	Limitations	References
*In vivo* (rabbit)	Rabbit glaucoma models	Elevated IOP; oxidative injury; anterior-segment stress	Oxidative stress; inflammatory responses	Changes in aqueous-humor oxidative markers; retinal and optic-nerve injury	IOP; AH/serum markers (MDA, GSH/GSSG, SOD, CAT, GPx, 8-OHdG); inflammatory cytokines; histology; optic-nerve morphology; PK/safety readouts	Larger eye enables surgical or pharmacologic intervention studies and repeat AH sampling	Limited genetic tools/resources than mouse models; disease course may not fully mirror chronic human glaucoma	( Panchal et al., 2017; Kanan et al., 2025 )
*In vivo* (non-human primate)	Primate glaucoma models	Chronic IOP elevation; long-term axonal stress	Axonal injury; neurodegenerative and glial pathways	ON degeneration; progressive visual dysfunction	IOP exposure; OCT RNFL and ONH parameters; ON axon counts; PERG or VEP; visual-field or behavioral data; tissue oxidative-injury markers	Closest ocular anatomy and optic-nerve-head architecture to human glaucoma	High cost; ethical constraints; small sample sizes and long study duration	( Yan et al., 2015; Fortune et al., 2016 )
Clinical studies	Human AH, blood/PBMCs, retina or ON tissue, imaging cohorts, and genetically patient subsets	Endogenous oxidative stress; mitochondrial decay; lipid/DNA oxidation; altered antioxidant defenses	DNA/lipid oxidation; redox imbalance; inflammatory-glial signaling; genotype-linked susceptibility	Biomarker validation; association with disease stage, progression, and treatment response	AH/serum/PBMC markers: 8-OHdG, MDA, protein carbonyls, GSH/GSSG, SOD, CAT, GPx; OCT RNFL/GCC; optic-disc/ONH parameters; IOP; visual-field indices (MD, VFI); genotype-biomarker correlation	Direct human relevance; supports translational biomarkers; incorporate inherited glaucoma subgroups for mechanistic insights	Limited tissue access; medication, age, and systemic comorbidities confound oxidative measurements; mostly are cross-sectional studies; incomplete genotype stratification	( Izzotti et al., 2003; Ferreira et al., 2004; Gherghel et al., 2005; Bagnis et al., 2012; Mohanty et al., 2022 )

Abbreviations: AH, aqueous humor; CAT, catalase; ERG, electroretinography; GCC, ganglion cell complex; GFAP, glial fibrillary acidic protein; GPx, glutathione peroxidase; GSH/GSSG, reduced/oxidized glutathione; IOP, intraocular pressure; MDA, malondialdehyde; NF-κB, nuclear factor kappa B; NOX, NADPH oxidase; OCT, optical coherence tomography; ON, optic nerve; ONH, optic nerve head; OCR/ECAR, oxygen consumption rate/extracellular acidification rate; PBMCs, peripheral blood mononuclear cells; PERG, pattern electroretinography; PK, pharmacokinetics; RGC, retinal ganglion cell; RNFL, retinal nerve fiber layer; ROS, reactive oxygen species; SOD, superoxide dismutase; tBHP, tert-butyl hydroperoxide; TEER, transepithelial electrical resistance; TM, trabecular meshwork; VEP, visual evoked potential; 8-OHdG, 8-hydroxy-2′-deoxyguanosine; *TBK1*, TANK-binding kinase 1; *MYOC*, myocilin; *OPTN*, optineurin; and WDR36, WD repeat domain 36.

**TABLE 5:** Research question-based selection of oxidative stress models in glaucoma.

Research objective	Recommended model(s)	Less suitable model(s)	Reason for selection	Key readouts/limitations
Acute ROS injury	H2O2/tBHP-treated TM or RGC cultures	Chronic animal models	Controlled dose and exposure time	Readouts: ROS, MitoSOX, viability Limitations: limited chronic relevance
TM oxidative damage	Primary/3D TM cultures; TM-on-chip	RGC-only models	Directly reflects anterior-segment outflow tissue	Readouts: ECM markers, ROS, outflow assays Limitations: limited systemic context
RGC vulnerability	Primary RGCs, iPSC-RGCs, retinal organoids	TM-only models	Direct assessment of neuronal oxidative injury	Readouts: RGC markers, neurites, apoptosis Limitations: limited ONH biomechanics
Chronic IOP-related stress	Microbead, EVC, hypertonic saline, DBA/2J	Acute ROS-treated cells	Captures pressure-related injury in intact eyes	Readouts: IOP, RGC counts, RNFL/OCT, ON axons Limitations: species variability
Genetic susceptibility	*MYOC*, *OPTN*, *TBK1*, *GLAST*, SOD1, iPSC models	Non-genetic acute injury models	Links oxidative stress to defined glaucoma pathways	Readouts: ROS, mitophagy, autophagy, RGC survival Limitations: genotype-specific
Human validation	AH, VH, PBMCs, donor eyes, surgical TM samples	Animal-only models	Provides direct clinical relevance	Readouts: 8-OHdG, MDA, GSH/GSSG, SOD/GPx Limitations: clinical confounders

Abbreviations: AH, aqueous humor; EVC, episcleral vein cauterization; GSH/GSSG, reduced/oxidized glutathione; iPSC, induced pluripotent stem cell; MDA, malondialdehyde; MitoSOX, mitochondrial superoxide indicator; ON, optic nerve; ONH, optic nerve head; PBMCs, peripheral blood mononuclear cells; RGC, retinal ganglion cell; RNFL, retinal nerve fiber layer; ROS, reactive oxygen species; TM, trabecular meshwork; VH, vitreous humor; 8-OHdG, 8-hydroxy-2′-deoxyguanosine.

### 4.3 Clinical evidence and human studies

Clinical studies in human populations demonstrate increased indicators of oxidative stress in certain glaucoma patient samples. There are several studies showing altered antioxidant profiles and increased oxidative damage products in AH (Ferreira et al., 2004; Goyal et al., 2014). Proteomic analysis of AH (Bagnis et al., 2012) supports that POAG molecular changes occur in response to oxidative stress. Furthermore, a meta-analysis verifies the constant significance of oxidative and anti-oxidative stress markers on chronic glaucoma in various cohorts (Benoist d’Azy et al., 2016). A systematic review and meta-analysis study of 22 case-control studies demonstrated that glaucoma patients exhibit an overall increase in oxidative stress markers (malondialdehyde) alongside altered antioxidant defenses (superoxide dismutase, glutathione peroxidase) in both serum and AH (Benoist d’Azy et al., 2016).

## 5 Molecular pathways of ROS-regulated glaucoma pathophysiology

The cellular vulnerability to oxidative damage in glaucoma is influenced by the balance between pro-oxidant pathways (NOX activation, mitochondrial failure, ferroptosis, and inflammatory signaling) and antioxidant defense systems (Nrf2/ARE signaling and glutathione regulation) (Wang et al., 2014; Fan Gaskin et al., 2021). These pathways are not isolated processes but a coordinated network of oxidative stress. In experimental models, whether the models are reflective of early redox modifications or later neurological damage relies on how each component proceeds. These pathways form an interconnected pathological network where early redox imbalances may contribute to late-stage structural damage.

### 5.1 Mitochondrial dysfunction

Mitochondrial dysfunction is the main source for the production and amplification of glaucoma ROS (Duarte, 2021; Ju et al., 2023). Mitochondrial DNA damage, reduced ATP, and impaired mitophagy are characteristics of glaucoma mitochondrial disease in the OHT and DBA/2J models. Oxidative stress commonly is associated with dysregulation of RGC mitochondrial homeostasis, which is reflected in abnormal mitochondrial dynamics such as enhanced Drp1-mediated fission (Kong et al., 2009; Kim et al., 2015). At the gene level, this manifests as upregulation of pro-fission genes (*DRP1, FIS1*) and downregulation of fusion genes (*MFN1, MFN2, OPA1*), a transcriptional pattern consistently observed in DBA/2J mice, suggesting a strong correlation between gene expression shifts to bioenergetic failure and RGC apoptosis (Ju et al., 2008).

These *in vitro* investigations of RGCs that were treated with mitochondrial poisons such as rotenone demonstrate early mitochondrial rupture prior to cell death. These findings provide credence to laboratory studies that indicate that oxidative stress has been linked to cause damage to mitochondria, which ultimately may contribute to apoptosis (Liu et al., 2019).

NAD^+^-dependent SIRT1 signaling has emerged as a therapeutic target for RGC neuroprotection, as RGC-selective AAV-mediated human *SIRT1* expression preserved visual function and reduced RGC loss in a mouse model of elevated IOP, while SIRT1-based therapy also modulated mitochondrial turnover pathways in retinal neurodegeneration models (Yue et al., 2023). A study shows that NAD-based neuroprotection preserves RGC structure and increases oxidative stress resistance. NAM supplementation maintains RGC density and inner retina function in glaucoma animals (Chiarugi, 2023; Visalli et al., 2026). Pharmacologically, mitochondrial neuroprotection strategies include NAD^+^ precursors (NAM, NR, NMN) that restore complex I activity, DRP1 inhibitors (e.g., Mdivi-1) that reduce excessive fission and preserve RGC mitochondrial networks (Kim et al., 2015), and mitophagy enhancers that promote clearance of damaged organelles and reduce feed-forward ROS amplification (Maddineni et al., 2026). These results show that mitochondrial instability is linked to oxidative stress in glaucomatous neurodegeneration and contributes to disease progression.

### 5.2 Nrf2/ARE antioxidant response

In glaucoma, oxidative stress stimulates the Nrf2/antioxidant response element (ARE) pathway, a main endogenous defense mechanism. OHT models indicate that higher IOP activates Nrf2 signaling in RGCs and glia, and is associated with increased ROS production. Nrf2 activation or overexpression protects RGCs against oxidative damage and susceptibility, while loss of Nrf2 exacerbates RGC susceptibility (Himori et al., 2013; Hvozda Arana et al., 2020; Naguib et al., 2021).

Nrf2 regulates the transcription of antioxidant and cytoprotective genes such as *H O -1, NQO1,* and *GCLC*, hence maintaining redox homeostasis under stress (Tonelli et al., 2018). However, chronic oxidative stress can paradoxically suppress Nrf2 through KEAP1-mediated degradation and NRF2 promoter hypermethylation observed in aged TM cells, providing a therapeutic rationale for KEAP1 inhibitors such as sulforaphane to restore endogenous antioxidant gene expression. Rodent OHT models, particularly those including *Nrf2* genetic manipulation, have provided significant insights into the timing and cell specificity of antioxidant responses in glaucoma (Himori et al., 2013; Brandes and Gray, 2020).

Furthermore, these models provide an opportunity to evaluate pharmacological Nrf2 and ARE-dependent transcription activators such as antioxidant and neuroprotective small molecules including sulforaphane (a KEAP1 cysteine modifier), dimethyl fumarate (an electrophilic ARE activator), and N-acetylcysteine (a glutathione [GSH]) precursor and indirect Nrf2 activator), each linked to protective effects in RGCs or TM cells in preclinical oxidative stress models (Fan Gaskin et al., 2021; Naguib et al., 2023). These results imply that Nrf2 signaling is an early adaptive response to oxidative stress. Long-term oxidative stress may reduce protective efficacy and contribute to RGC and TM cell damage.

### 5.3 NOX enzymes and superoxide production

NADPH oxidases (NOX enzymes) and ROS production are becoming progressively implicated in glaucomatous neurodegeneration. Experimental studies have shown that NOX activation is closely linked to generation of ROS and associated with RGC death (Dvoriantchikova et al., 2012). NOX isoforms, in particular NOX2 and NOX4, have also been shown to be involved in oxidative stress responses in models of retinal and glaucoma damage (Duarte, 2021; Liao et al., 2023). Both *NOX2* (encoded by *CYBB*) and NOX4 are transcriptionally upregulated in glaucomatous TM tissue via NF-κB and HIF-1α, which has been linked to a self-reinforcing oxidative loop. The regulatory subunit p22phox (encoded by *CYBA*) is required for NOX4 complex assembly and represents a candidate molecular target for interrupting upstream ROS generation. Redox cross-talk between NOX-derived superoxide and mitochondrial dysfunction may augment oxidative damage in RGCs. NOX activation is a possible therapeutic target for oxidative stress, which chronologically parallels or precedes mitochondrial damage (Fan Gaskin et al., 2021). Selective NOX4 inhibition with GLX351322 significantly reduces retinal ROS, attenuates microglial activation, and protects RGCs in acute OHT models (Liao et al., 2023), while pan-NOX inhibitors such as apocynin reduce superoxide-driven NF-κB activation and RGC loss in ischemia-reperfusion models. Isoform selectivity is important, as NOX2 is strongly associated with neuroinflammatory ROS in microglia whereas NOX4 correlates with cell-autonomous mitochondrial ROS in TM and RGCs. Targeted studies, like genetic or pharmacological NOX inhibition before IOP elevation, are required to determine whether NOX activation contributes to mitochondrial dysfunction and RGC depletion (Fan Gaskin et al., 2021).

### 5.4 Glutathione depletion

Glutathione depletion is a loss of an antioxidant defense pathway, not a key pro-oxidant mechanism. GSH is a significant redox buffer in the cell that detoxifies ROS and maintains antioxidant protection in TM cells and RGCs. Reduced GSH levels and compromised antioxidant defenses, including decreased SOD and GPx activity, are observed in experimental models of glaucoma and clinical samples, indicating impaired redox regulation in the face of persistent glaucomatous stress (Izzotti et al., 2003; Gherghel et al., 2005; Chrysostomou et al., 2013; Fan Gaskin et al., 2021).

Thus, GSH depletion correlated with an impaired antioxidant defense system and the acceleration of pro-oxidant pathways, including mitochondrial ROS generation, lipid peroxidation, and inflammatory oxidative signaling. *In vitro* TM and retinal models provide controlled systems for evaluating therapies aimed at restoring GSH levels or increasing the activity of antioxidant enzymes. Rodent and rabbit OHT models show progressive antioxidant failure in chronic glaucomatous neurodegeneration (Maher and Hanneken, 2005; Bagnis et al., 2012).

GSH depletion also promotes ferroptosis, a downstream oxidative cell-death process involving inadequate detoxification of lipid peroxides and iron-dependent lipid peroxidation (Qin et al., 2026). Thus, GSH depletion is a tipping point associated with a transition from adaptive antioxidant responses to irreversible oxidative damage and neurodegeneration (Chrysostomou et al., 2013; Fan Gaskin et al., 2021). Therapeutically, the antioxidant defense can be enhanced by restoring GSH with N-acetylcysteine (NAC) or glutathione ethyl ester (GSH-EE). Ferroptosis inhibitors, such as ferrostatin-1 and liproxstatin-1, inhibit the lipid peroxidation cascade linked to ferroptotic RGC death (Sano et al., 2019; Guo et al., 2022; Yan et al., 2024).

### 5.5 Pathways of apoptosis in RGC death

Elevated IOP was linked to RGC apoptosis in early experimental glaucoma studies (Quigley et al., 1995). This process is mediated by mitochondrial pathways and extrinsic death receptor signaling. Experimental glaucoma has shown a clear correlation between RGC degeneration and Fas/FasL signaling, and Fas signaling blockade either genetically or pharmacologically has shown neuroprotective properties *in vivo* (Krishnan et al., 2016; 2019). Brimonidine activates PI3K/Akt and ERK1/2 pro-survival pathways in RGCs independently of IOP lowering, while BDNF/TrkB augmentation via AAV2-BDNF gene delivery or TrkB agonists suppresses Bax-mediated apoptosis and preserves RGC axons in rat experimental OHT models. Brimonidine may support RGC survival through regulated PI3K/Akt and ERK1/2 activation independently of IOP lowering, although dysregulated or prolonged activation of these pathways can also contribute to apoptotic stress; BDNF/TrkB augmentation suppresses Bax-mediated apoptosis and preserves RGC axons in OHT models (Kimura et al., 2016; Wójcik-Gryciuk et al., 2020). Likewise, TNF-α signaling is related to glaucomatous RGC destruction. Soluble TNF-α from glia is associated with RGC death in glaucoma models, and TNF-α/TNFR1 signaling may contribute to RGC dysfunction and apoptosis (Cueva Vargas et al., 2015; Cheng et al., 2021).

In models of glaucoma, apoptosis is also associated with caspase activation, mitochondrial dysfunction, inflammatory signaling, and PI3K/Akt impairment (Nie et al., 2018).

*In vitro* cell culture systems have been used to assess apoptosis inhibitors or survival pathway activators, and *in vivo* models have shown RGC protection with specific therapies such as Fas receptor antagonism (Krishnan et al., 2016; 2019).

Apoptotic pathways are important convergence sites of oxidative, inflammatory, and mitochondrial stress signals in glaucoma and continue to be relevant targets for mechanistic inquiry and neuroprotective medication development (Levkovitch-Verbin, 2015; Basavarajappa et al., 2023; Zhao et al., 2023).

### 5.6 Inflammatory pathways driven by oxidative damage

Oxidative stress and neuroinflammation are mutually promoting in glaucomatous neurodegeneration (Rolle et al., 2021; Tezel, 2022). Microglial activation is strongly associated with RGC degeneration in models of acute and chronic glaucoma. RGC degeneration has been linked to inflammasome activation, including NLRP3 signaling and IL-1β production (Chi et al., 2014; Coyle et al., 2021). Mitochondrial damage and the release of signals are linked to NLRP3 inflammasome activation and increased levels of IL-1β and IL-18 in OHT and DBA/2J mice (Coyle et al., 2021; Li et al., 2019). Activated microglia and astrocytes release pro-inflammatory cytokines and reactive oxygen species, perpetuating oxidative and inflammatory damage (Wei et al., 2019). Recapitulation of inflammatory reactions *in vitro* in retinal and mixed glial cultures may be used to study the NF-κB signaling pathway in a controlled setting and to evaluate anti-inflammatory treatments, such as inflammasome inhibition. (Duarte, 2021; Jassim et al., 2021). A positive feedback loop between oxidative stress and inflammation drives ongoing neurodegeneration in glaucoma (Coyle et al., 2021).

### 5.7 Hypoxia/reoxygenation-induced oxidative stress pathways

Hypoxia/reoxygenation is a well-established paradigm to imitate the oxidative stress associated with ischemia and yields important insights into ROS-driven neuronal mechanisms in glaucoma (Osborne et al., 2004). The sudden rise in ROS, in particular mitochondrial ROS, following hypoxia and reoxygenation is associated with oxidative damage and cellular dysfunction. Redox-sensitive probes may be used to detect mitochondrial superoxide production and redox imbalance under H/R conditions. These data demonstrate enhanced formation of hydrogen peroxide, hydroxyl radical, and superoxide upon reperfusion (Robinson et al., 2006).

Besides the generation of ROS, hypoxic signaling pathways are activated, such as the stabilization of hypoxia-inducible factor-1α (HIF-1α), which is indicative of the cellular adaptation to low amounts of oxygen. Reoxygenation increases oxidative injury, which is closely linked to membrane damage and cell death, as shown by lactate dehydrogenase (LDH) release.

These activities are indicative of the cross-talk between hypoxia, mitochondrial failure, and oxidative stress and demonstrate how ischemia contributes to damage in RGCs. H/R systems aid in understanding vascular and metabolic contributors to glaucoma and evaluating oxidative stress-targeted treatments.

These pathways are also potential therapeutic targets, where drug-based or interventional strategies may be matched to the underlying disease mechanisms. Examples include NOX inhibitors that limit ROS generation, Nrf2 activators that increase antioxidant defenses, and mitochondrial-targeted treatments such as NAD^+^ supplements that promote cellular resilience.

## 6 Discussion

This review summarizes experimental and clinical evidence linking oxidative stress to glaucoma pathophysiology across *in vitro*, *in vivo*, and human sample-based models. By comparing these systems, we aimed to clarify how model selection influences interpretation of ROS-related mechanisms and translational relevance. Overall, ROS are associated with TM dysfunction, IOP dysregulation, and RGC degeneration in several glaucoma models and patient-derived samples. Across *in vitro*, *in vivo,* and human sample-based studies, oxidative stress has been associated with mitochondrial dysfunction, impaired antioxidant defenses, neuroinflammation, and apoptotic cell death pathways (Rohowetz et al., 2018).

Oxidative stress findings should be interpreted with each model’s strengths and limits in mind. Comparing models helps show which findings are model-specific and which may apply to human glaucoma. Acute *in vitro* ROS exposure models are reproducible and useful for mechanistic screening; however, they may overestimate injury when compared to chronic glaucoma. This is due to the fact that short-term oxidative exposure does not fully reproduce the slow and multifactorial nature of glaucomatous neurodegeneration (Fomo et al., 2024). Animal ocular hypertension models can capture pressure-related damage to the retina and optic nerve. However, results can be affected by inflammation from the procedure, species differences, and variable IOP elevation (Bugara et al., 2024). Human biospecimens provide direct clinical relevance, although age, medication, systemic disease, and sample timing may affect oxidative stress measurements. Therefore, findings are strongest when similar oxidative markers or protective effects are reproduced across complementary *in vitro*, *in vivo*, and human systems. Importantly, increased ROS levels or oxidative biomarkers should not always be interpreted as proof of causality. Stronger causal inference requires interventional, genetic, or rescue studies. These studies show that changing oxidative stress pathways may modulate or affect RGC survival, TM function, or optic nerve outcomes (Benoist d’Azy et al., 2016).

**FIGURE 2:**
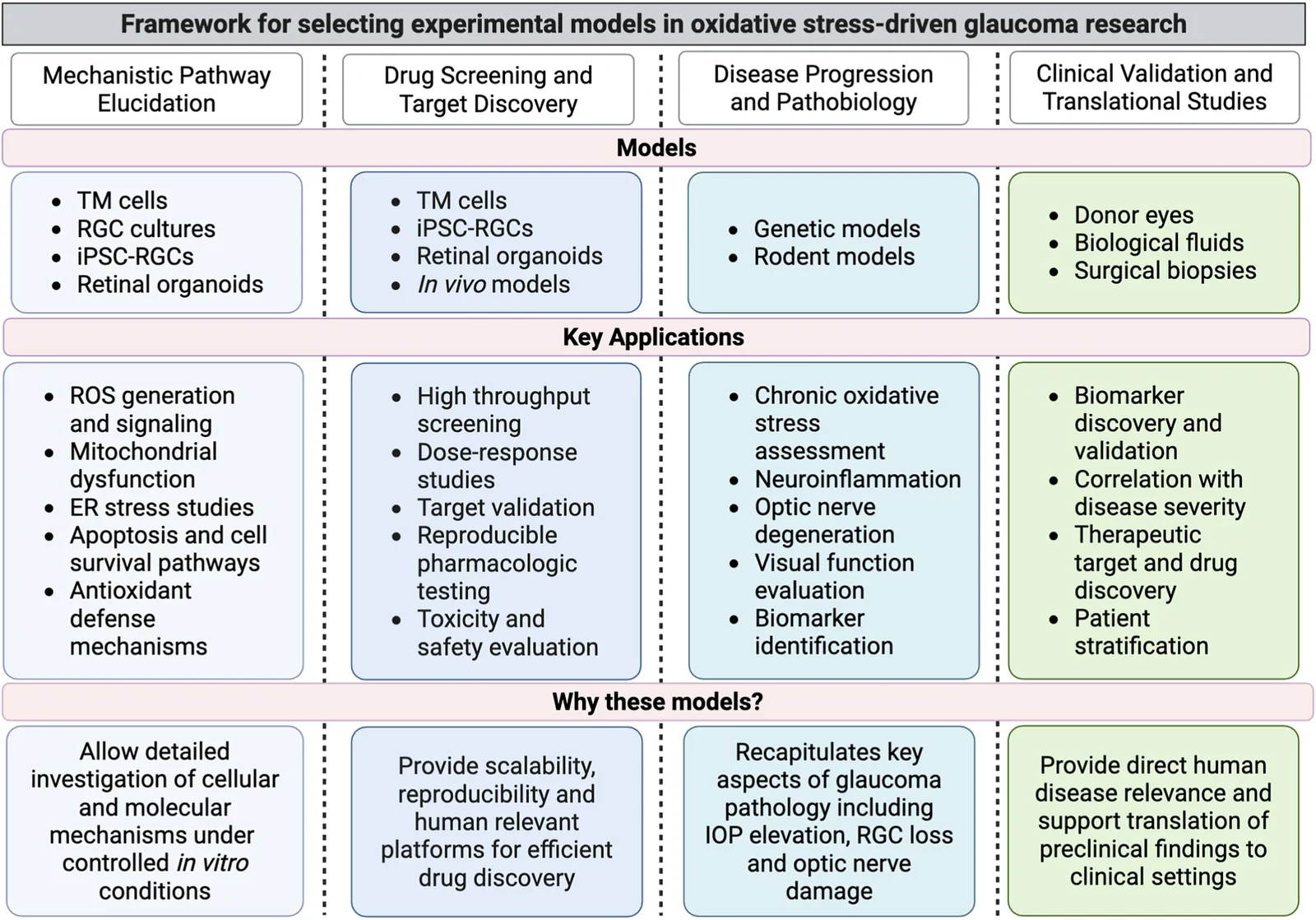
Framework for selecting experimental models in oxidative stress-driven glaucoma research. This figure summarizes model-selection considerations for oxidative stress-focused glaucoma research according to research purpose. Trabecular meshwork cells, retinal ganglion cell cultures, induced pluripotent stem cell-derived retinal ganglion cells, and retinal organoids are useful for controlled *in vitro* studies of cellular and molecular mechanisms. Cell-based systems, retinal organoids, and selected *in vivo* models can support drug screening, target discovery, pharmacologic testing, and toxicity assessment. Genetic and animal models are useful for studying chronic oxidative stress, neuroinflammation, optic nerve degeneration, visual function, biomarker identification, and disease progression. Human donor eyes, biological fluids, and surgical biopsies are most relevant for biomarker validation, patient stratification, therapeutic target assessment, and clinical translation. Abbreviations: TM, trabecular meshwork; RGC, retinal ganglion cell; iPSC-RGCs, induced pluripotent stem cell-derived retinal ganglion cells; ROS, reactive oxygen species; ER, endoplasmic reticulum. Created with BioRender.

High-resolution *in vitro* models enable in-depth investigation of ROS-associated alterations in TM cells, RGCs, and glial populations. These systems also include iPSC-derived RGCs and ROs, as summarized in Figure 2. The main strength of these systems lies in their scalability and experimental control. This allows high-throughput screening and focused study of downstream pathways. 3D cultures have improved the physiological relevance of these systems. They also allow controlled study of redox-sensitive phenotypes, genetic vulnerability, and treatment response. They are also useful for high-throughput screening, dose-response studies, reproducible pharmacologic testing, and toxicity or safety evaluation. However, their primary limitation remains an inability to imitate the chronic, multicellular, and vascular components of glaucoma.

*In vivo* models also include systemic complexity and disease development over time. In chronic ocular hypertension models, including microbead occlusion, episcleral vein cauterization, and genetically predisposed mice, increased ROS production has been observed before or alongside neurodegenerative changes (Williams et al., 2017; Bugara et al., 2024). These findings suggest a temporal association between oxidative stress, mitochondrial dysfunction, impaired antioxidant responses, and progressive RGC depletion; however, they do not by themselves prove that oxidative stress is the primary upstream driver of glaucoma progression. Furthermore, a persistent limitation of these models is that species-specific anatomical differences and acute procedural IOP spikes can limit clinical translation. A stronger causal interpretation requires interventional, genetic, or rescue-based studies showing that modulation of oxidative stress pathways modulates TM function, RGC survival, or optic nerve outcomes (Inman et al., 2013; Williams et al., 2017). This model-based view helps identify which systems are best suited for specific oxidative stress questions in glaucoma.

Laboratory models of glaucoma are providing increasing evidence of oxidative damage, mitochondrial abnormalities, and redox imbalance in glaucomatous eyes. These findings are corroborated by data from human ocular tissues and clinical samples. Biomarkers can be obtained from clinical sources such as biological fluids, surgical biopsies, and donor eyes. These biomarkers can be used for validation, correlation with treatment, and disease stratification. Human clinical samples are highly relevant, but they also have important limitations. Patient age, treatment history, disease stage, medical history, and sample timing can all affect the results. These factors make it difficult to follow real-time disease progression from clinical samples alone. The fact that they are consistently found across different model systems highlights the translational significance of ROS-associated pathways as well as the necessity of selecting models based on their mechanisms.

Bridging early oxidative stress and permanent neuronal death requires integrating mechanistically precise *in vitro* systems with clinically relevant *in vivo* models. To develop successful therapeutics that target ROS generation, mitochondrial integrity, antioxidant signaling, and oxidative-inflammatory feedback loops, candidate molecules must be matched to the model system that is most mechanistically applicable. Potential therapeutic alternatives include NAC, ferrostatin-1, Nrf2 activation, NOX inhibition, mitochondrial support, and neurotrophic drugs (BDNF, brimonidine) (Fan Gaskin et al., 2021).

In conclusion, the involvement of oxidative stress in the pathophysiology of glaucoma establishes a connection between biological pathways and disease progression. By synthesizing these data, this review reinforces a strategic framework for model selection, ensuring that future study designs explicitly match their clinical objectives against the specific strengths and limitations of the selected platform. Future neuroprotective strategies need not only go beyond IOP reduction but also need to be aligned with the molecular mechanisms of glaucomatous neurodegeneration, such as ROS-associated pathways (Visalli et al., 2026). This is consistent with a neuroprotective model of glaucoma in which oxidative stress correlated with cellular injury, damage, and depletion of trophic support at the synapse. This method shows that synaptic maintenance and neurotrophic modulation are exciting prospects for therapy (Kimura et al., 2016; Mallone et al., 2020; Wójcik-Gryciuk et al., 2020).

Importantly, the pairing of focused pharmacologic treatments with suitable experimental models will be important to improve the translational efficacy of oxidative stress–based therapeutics in glaucoma. This schematic will help researchers to choose experimental models to study oxidative stress pathways in glaucoma according to study aims. Models such as TM cells, RGC cultures, iPSC-derived RGCs, and ROs are suitable for mechanistic studies, oxidative stress signaling analysis, mitochondrial dysfunction, ER stress, apoptosis, and high-throughput drug screening in well-controlled experimental conditions. *In vivo* models like microbead-induced OHT, EVC, genetic, and traditional models in mice/rats can be utilized to investigate ongoing oxidative stress, neuroinflammation, ON degeneration, and disease development. Human clinical samples, including donor eyes, aqueous and vitreous fluids, blood-derived samples, PBMCs, and surgical biopsies, can be used to identify oxidative damage biomarkers, validate experimental findings, and examine their association with glaucoma severity. Figure 2 displays the balance in model selection between mechanistic resolution, physiological relevance, scalability, translational applicability, and experimental feasibility with respect to the biological issue, while Table 5 expands this framework by identifying recommended and less suitable models for specific oxidative stress-related glaucoma research questions.

## 7 Future directions

Future studies should seek to integrate transcriptomic, proteomic, and spatial analyses to further understand cell-type-specific oxidative stress responses in glaucoma. High-throughput screening and targeted modulation of ROS-regulated pathways, such as NOX signaling, mitochondrial function, and antioxidant defenses, will allow a more precise investigation of patient-specific susceptibility and variant-driven mechanisms using iPSC-derived retinal and organoid models. Vascular and immunological components should be included in the next-generation of platforms, such as *ex vivo*, organ-on-a-chip, and immune retinal co-culture systems, to increase the physiological relevance and study the ROS-driven inflammatory interactions.

## 8 Conclusion

This review summarizes the use of experimental and clinical models to study oxidative stress in glaucoma. No single model fully reflects human disease. Therefore, the choice of model should depend on the specific research question, the disease stage being studied, and how the findings will be translated. Using more than one model may help confirm oxidative stress findings and guide future neuroprotective studies in glaucoma.

## Glossary

2D: two-dimensional
3D: three-dimensional
4-HNE: 4-hydroxynonenal
8-OHdG: 8-hydroxy-2′-deoxyguanosine
AAV: adeno-associated virus
*ABCA1*: ATP-binding cassette transporter A1
AH: aqueous humor
ARE: antioxidant response element
ATP: adenosine triphosphate
ATOH7: atonal bHLH transcription factor 7
*BAX*: BCL2-associated X protein
Bcl-2: B-cell lymphoma 2
BDNF: brain-derived neurotrophic factor
BiP: binding immunoglobulin protein
Brn3a: brain-specific homeobox/POU domain protein 3A
CAT: catalase
*CDKN2B-AS1*: cyclin-dependent kinase inhibitor 2B antisense RNA 1
CHOP: C/EBP homologous protein
DCFDA: 2′,7′-dichlorofluorescin diacetate
DHE: dihydroethidium
DNA: deoxyribonucleic acid
DBA/2J: dilute brown non-agouti 2 Jackson mouse strain
ECM: extracellular matrix
ER: endoplasmic reticulum
ERG: electroretinography
ERK: extracellular signal-regulated kinase
ETC: electron transport chain
EVC: episcleral vein cauterization
GCC: ganglion cell complex
GFAP: glial fibrillary acidic protein
*GLAST*: glutamate/aspartate transporter
GPx: glutathione peroxidase
GRP78: glucose-regulated protein 78
GSH/GSSG: reduced/oxidized glutathione
GWAS: genome-wide association study
**H**_**2**_ **O**_**2**_: hydrogen peroxide
H/R: hypoxia/reoxygenation
HIF-1α: hypoxia-inducible factor 1-alpha
HRT: Heidelberg Retina Tomograph
IL-1β: interleukin-1 beta
IL-6: interleukin-6
IL-18: interleukin-18
IOP: intraocular pressure
iPSC: induced pluripotent stem cell
iPSC-RGC: induced pluripotent stem cell-derived retinal ganglion cell
iPSC-TM: induced pluripotent stem cell-derived trabecular meshwork cell
JC-1/TMRE: mitochondrial membrane potential indicators JC-1/tetramethylrhodamine ethyl ester
LC: lamina cribrosa
LC3: microtubule-associated protein 1 light chain 3
MAPK: mitogen-activated protein kinase
MD: mean deviation
MDA: malondialdehyde
MitoSOX: mitochondrial superoxide indicator
*MYOC*: myocilin
NADPH: nicotinamide adenine dinucleotide phosphate
NF-κB: nuclear factor kappa B
NHP: non-human primate
NMDA: N-methyl-D-aspartate
NOX: NADPH oxidase
Nrf2: nuclear factor erythroid 2-related factor 2
NTG: normal-tension glaucoma
OCT: optical coherence tomography
OCR/ECAR: oxygen consumption rate/extracellular acidification rate
OHT: ocular hypertension
ON: optic nerve
ONC: optic nerve crush
ONH: optic nerve head
OPA1: optic atrophy 1
*OPTN*: optineurin
p62: sequestosome 1
PBMCs: peripheral blood mononuclear cells
PERG: pattern electroretinography
POAG: primary open-angle glaucoma
PSD-95: postsynaptic density protein 95
RBPMS: RNA-binding protein with multiple splicing
RGC: retinal ganglion cell
RNFL: retinal nerve fiber layer
RO: retinal organoid
ROS: reactive oxygen species
*SIRT1*: sirtuin 1
*SIX6*: SIX homeobox 6
*SLC1A3*: solute carrier family 1 member 3
SOD: superoxide dismutase
*SOD1*: superoxide dismutase 1
*TBK1*: TANK-binding kinase 1
tBHP: tert-butyl hydroperoxide
TEER: transepithelial electrical resistance
TGF-β2: transforming growth factor beta 2
TM: trabecular meshwork
TNF-α: tumor necrosis factor alpha
TNFR1: tumor necrosis factor receptor 1
TrkB: tropomyosin receptor kinase B
TUNEL: terminal deoxynucleotidyl transferase dUTP nick end labeling
*TXNRD2*: thioredoxin reductase 2
UPR: unfolded protein response
VEP: visual evoked potential
VF: visual field
VFI: visual field index
VH: vitreous humor
WDR36: WD repeat domain 36
XBP1: X-box binding protein 1
α-SMA: alpha-smooth muscle actin
